# Neurodevelopmental deficits and cell-type-specific transcriptomic perturbations in a mouse model of *HNRNPU* haploinsufficiency

**DOI:** 10.1371/journal.pgen.1010952

**Published:** 2023-10-02

**Authors:** Sarah A. Dugger, Ryan S. Dhindsa, Gabriela De Almeida Sampaio, Andrew K. Ressler, Elizabeth E. Rafikian, Sabrina Petri, Verity A. Letts, JiaJie Teoh, Junqiang Ye, Sophie Colombo, Yueqing Peng, Mu Yang, Michael J. Boland, Wayne N. Frankel, David B. Goldstein

**Affiliations:** 1 Institute for Genomic Medicine, Columbia University Irving Medical Center, New York, New York, United States of America; 2 Department of Genetics and Development, Columbia University Irving Medical Center, New York, New York, United States of America; 3 Department of Pathology & Immunology, Baylor College of Medicine, Houston, Texas, United States of America; 4 Jan and Dan Duncan Neurological Research Institute of Texas Children’s Hospital, Houston, Texas, United States of America; 5 Mouse Neurobehavioral Core Facility, Columbia University Irving Medical Center, New York, New York, United States of America; 6 Department of Biochemistry and Molecular Biophysics, Columbia University Irving Medical Center, New York, New York, United States of America; 7 Zuckerman Mind Brain and Behavior Institute, Columbia University, New York, New York, United States of America; 8 Department of Pathology and Cell Biology, Columbia University Irving Medical Center, New York, New York, United States of America; 9 Department of Neurology, Columbia University Irving Medical Center, New York, New York, United States of America; HudsonAlpha Institute for Biotechnology, UNITED STATES

## Abstract

Heterozygous *de novo* loss-of-function mutations in the gene expression regulator *HNRNPU* cause an early-onset developmental and epileptic encephalopathy. To gain insight into pathological mechanisms and lay the potential groundwork for developing targeted therapies, we characterized the neurophysiologic and cell-type-specific transcriptomic consequences of a mouse model of *HNRNPU* haploinsufficiency. Heterozygous mutants demonstrated global developmental delay, impaired ultrasonic vocalizations, cognitive dysfunction and increased seizure susceptibility, thus modeling aspects of the human disease. Single-cell RNA-sequencing of hippocampal and neocortical cells revealed widespread, yet modest, dysregulation of gene expression across mutant neuronal subtypes. We observed an increased burden of differentially-expressed genes in mutant excitatory neurons of the subiculum—a region of the hippocampus implicated in temporal lobe epilepsy. Evaluation of transcriptomic signature reversal as a therapeutic strategy highlights the potential importance of generating cell-type-specific signatures. Overall, this work provides insight into *HNRNPU*-mediated disease mechanisms and provides a framework for using single-cell RNA-sequencing to study transcriptional regulators implicated in disease.

## Author summary

The brain relies on strict regulation of gene expression for proper functioning. Mutations in genes that influence the expression of other genes are linked to neurological conditions such as epilepsy, autism, intellectual disability, and neurodegenerative disorders. Identifying targeted therapies for these genetic causes of disease are increasingly difficult since they often lead to a wide array of molecular and cellular effects from the widespread disturbance in gene expression. One potential approach is to identify therapies that shift the abnormal gene expression signature to a more normal state. To explore this ‘transcriptome-guided’ approach, we characterized the consequences of the loss of one copy of the gene expression regulator, *HNRNPU*, from the molecular to the whole organism level using a genetic mouse model. We generated disease-associated gene expression signatures and compared them to signatures obtained from cells treated with different drugs to pinpoint compounds with the potential to rescue abnormal gene expression.

## Introduction

Given their functional complexity, high metabolic demands, and extensive diversity, it is unsurprising that neurons rely particularly on the strict regulation of gene expression. In fact, mutations in genes that cause gene expression dysregulation, including chromatin modifiers [1,2], transcription factors [3,4] and RNA-binding proteins [5,6], are a well-described cause of neurodevelopmental disease, including epilepsy and autism. Considering mutations in this class of molecules often lead to the dysregulated expression of thousands of genes within vulnerable cell types, pinpointing therapeutically tractable disease mechanisms is especially challenging. These pleiotropic effects necessitate the use of high-resolution phenotyping assays. One powerful approach is single cell RNA-sequencing (scRNAseq), which allows the identification of cell-type-specific gene expression changes in disease-associated tissues. Having such granular insight into transcriptomic dysregulation may not only inform on cell and tissue-specific disease mechanisms but could also serve as an opportunity for drug development through a “transcriptome reversal” approach aimed at identifying compounds capable of reverting disease-associated gene expression signatures to a normal state. This approach has been applied in cancer and other complex diseases [7–9], but has recently also been adopted for neurological disorders that have notoriously been difficult to drug [10,11]. Here, using scRNAseq and a genetic mouse model of *HNRNPU* haploinsufficiency, we extend this transcriptome-guided precision medicine approach to a monogenic neurodevelopmental disease.

*HNRNPU* (heterogeneous nuclear ribonuclear protein U) encodes a ubiquitously-expressed, DNA- and RNA-binding protein, hnRNP U, that localizes to the nucleus [12,13], where it mediates gene expression through transcription initiation and elongation [14–19], pre-mRNA processing [20,21] and chromatin organization [22,23]. We and others have reported *de novo* loss-of-function variants [24–28] and microdeletions [29,30] encompassing *HNRNPU* in pediatric patients with a severe, and often treatment refractory, developmental and epileptic encephalopathy (DEE) characterized by early-onset epilepsy, moderate to severe developmental delay, autistic features, structural brain abnormalities, hypotonia, short stature and variable renal and cardiac abnormalities. *HNRNPU* is essential for mammalian development as lethality results by embryonic day 11.5 in mice carrying homozygous hypomorphic mutations [31]. Furthermore, homozygous pathogenic mutations have yet to be reported in humans [32]. Conditional loss of *Hnrnpu* in mouse cardiomyocytes was also associated with a lethal dilated cardiomyopathy and widespread transcriptional and splicing dysregulation including known cardiomyopathy disease genes [21]. However, the transcriptomic and physiologic effects of *Hnrnpu* haploinsufficiency in the brain have yet to be characterized.

Here we assess the neurophysiological consequences and face validity of an *Hnrnpu* mouse disease model using *in vivo* developmental, morphological, electrophysiological, and behavioral studies. We also perform a comprehensive cell-type- and brain region-specific characterization of reduced *Hnrnpu* levels on gene expression using scRNAseq at a single postnatal time point. Using these data, we generate cell-type-specific disease expression signatures and identify vulnerable cell types in the mutant mouse brain. We then compare these signatures to publicly available gene expression signatures of cells treated with small molecules to identify compounds that correct disease-associated transcriptomic changes towards a normal state. Overall, this work provides a framework for high-resolution phenotyping of models of transcriptome-mediated diseases and outlines important considerations for the future development of targeted therapies for *HNRNPU* DEE.

## Results

### Generation of an *Hnrnpu* knockout mouse model

HnRNP U protein expression is widespread in the brain, yet particularly concentrated within the cerebellum, hippocampus and neocortex [33]. In mouse primary cell cultures derived from the neocortex and hippocampus, hnRNP U co-stained with the neuronal marker Map 2, and markers of neuronal subtypes including inhibitory neurons (Gad67) as well as cortical pyramidal neurons of deep (Ctip2) and superficial (Satb2) lamina (S1A–S1D and S2A–S2D Figs). HnRNP U also co-stained with the astrocyte marker Gfap (S1E and S2E Figs). For all cells examined, hnRNP U expression appeared confined to the nucleus, as previously reported [12,13].

**Fig 1:**
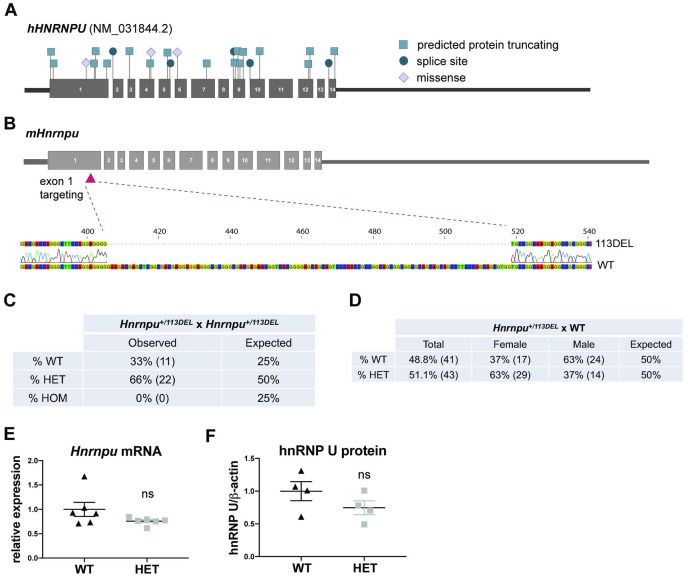
Generation of a mouse model of *HNRNPU* haploinsufficiency. **(A)** Location of predicted pathogenic variants, including protein truncating, splice site and missense (listed in S1 Table) across all 14 coding exons of human *HNRNPU* (NM_031844.2). Non-coding sequence not drawn to scale. **(B)** Location of the CRISPR-induced 113-bp deletion in exon 1 of mouse *Hnrnpu*. **(C)** Observed versus expected Mendelian ratios from *Hnrnpu*^+/113DEL^ intercrosses (10 litters) at PND0 (Chi-square p = 4x10^-3^). (**D)** Observed versus expected ratios from *Hnrnpu*^+/113DEL^ x WT crosses at PND0 (16 litters) with male and female breakdown (Chi-square p-values: total = 0.82, males = 0.10 and females = 0.08). PND = postnatal day 0. **(E)** *Hnrnpu* expression relative to *Cyc1* reference gene (Welch’s t-test p = 0.15 (two-tailed, t = 1.65, df = 5)). **(F)** Mouse hnRNP U protein expression quantified by densitometry and normalized to mouse β-actin (Unpaired t-test p = 0.21 (two-tailed, t = 1.4, df = 6)). Error bars = SEM

Most pathogenic mutations in *HNRNPU* are loss-of-function (Fig 1A and S1 Table). We therefore targeted exon 1 of mouse *Hnrnpu—*a region in which at least five premature truncating mutations were identified in human patients—to induce a constitutive out-of-frame deletion (Fig 1A and 1B and S1 Table). A founder containing a heterozygous 113-bp deletion (herein referred to as *Hnrnpu*^*+/*113DEL^ or HET) with resulting premature stop codon in exon 2 was identified and used to expand the line, which was subsequently maintained on an inbred C57BL/6NJ background (Figs 1B and S3A). As expected, *Hnrnpu*^+/113DEL^ intercrosses did not produce any viable homozygous mutant progeny, consistent with embryonic lethality (Fig 1C). For heterozygous mutant mice, there was no significant sex-specific differences in viability at birth (Fig 1D).

Evaluation of both mRNA and protein levels obtained from cerebral cortex revealed a decrease in *Hnrnpu* expression of approximately 20–25% in *Hnrnpu*^+/113DEL^ mice (Figs 1E, 1F, and S3B). Although this difference was not statistically significant due to insufficient power, cortical single-cell RNA-sequencing (scRNA-seq) also revealed a significant 26% reduction in *Hnrnpu* RNA expression (Wilcoxon p = 3.2x10^-39^). Furthermore, we did not detect a truncated form of hnNRP U when using an antibody that binds N-terminal to the deletion breakpoint (S3C Fig). In line with these findings in mice, two isogenic human induced pluripotent stem cell (hiPSC)-derived cortical organoid models containing heterozygous loss-of-function variants also showed an insignificant reduction of roughly 25% *HNRNPU* mRNA and protein compared to an isogenic control [34]. These results suggest that near normal WT expression of *HNRNPU* may be necessary for development, and hint at the presence of compensatory mechanisms in response to loss-of-function variants.

### No overt brain abnormalities in mutant *Hnrnpu* mice

Patients with *HNRNPU* mutations present with a variety of central nervous system abnormalities, including neuronal migration defects, enlarged lateral ventricles, corpus callosum defects, delayed myelination and mild holoprosencephaly [26–28]. Furthermore, the human cortical organoid models reported by Ressler and colleagues showed a significant size reduction [34] and an independent mouse model containing a homozygous truncating variant also demonstrated impaired cortical development [35].

In the current study of heterozygous mutants, we did not observe differences in brain size and corpus callosum morphology compared to WT at postnatal day 0 (PND0) (S4A Fig). Further examination of *Hnrnpu*^+/113DEL^ brains also showed no significant change in cortical thickness and hippocampal width (S4B Fig). Additional high-resolution studies capable of detecting subtler morphological abnormalities are warranted.

### *Hnrnpu*^+/113DEL^ pups show global developmental delay

Patients with *HNRNPU*-associated DEE frequently demonstrate axial hypotonia along with moderate to severe developmental delay, primarily manifesting as delayed motor skills and severe speech impairment [26–28]. We therefore evaluated early physical and sensorimotor development including growth, righting reflex, negative geotaxis and vertical screen hold in the first two weeks of life.

**Fig 2:**
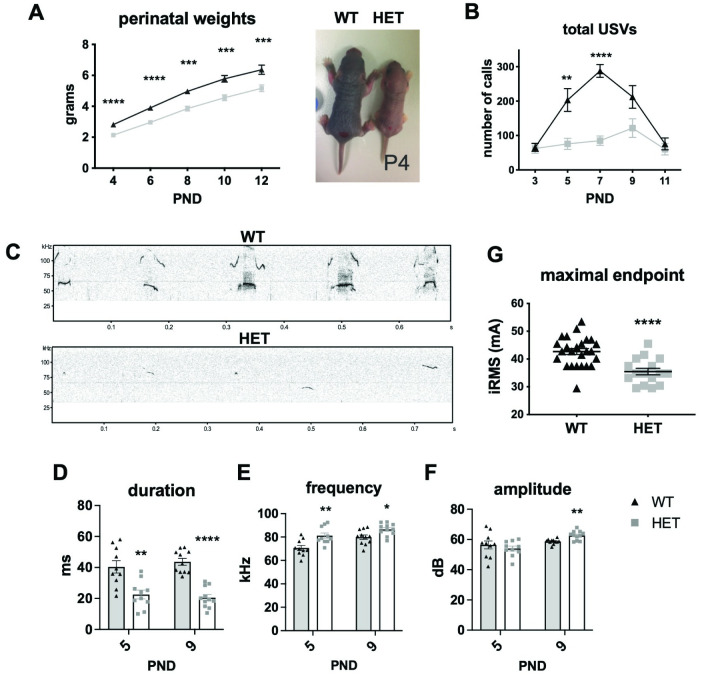
*Hnrnpu*^+/113DEL^ mouse phenotypes. **(A)** Body weight in the perinatal period (n = 16 WT, 16 HET). Permuted Mann Whitney U (MWU) p-values: PND4-6< 1x10^-4^, PND8 = 2x10^-4^, PND10 = 1x10^-4^, PND12 = 1.5x10^-3^. Representative image of a PND4 WT and *Hnrnpu*^*+/113DEL*^ pup. **(B)** Total number of pup calls over a 3 min time interval (n = 17 WT, 16 HET). Permuted MWU p-values: PND3 = 0.61, PND5 = 6.8x10^-3^, PND7< 1x10^-4^, PND9 = 0.06, PND11 = 0.49. (**C**) Representative WT and HET image of an ultrasonic vocalization spectrograph. **(D)** Average pup call duration. Permuted MWU p-values: PND5 = 2.1x10^-3^, PND9< 1x10^-4^. **(E)** Average peak frequency (i.e. pitch). Permuted MWU p-values: PND5 = 6.9x10^-3^, PND9 = 0.02. **(F)** Average peak amplitude (i.e. loudness). Permuted MWU p-values: PND5 = 0.43, PND9 = 3.3x10^-3^. For all qualitative USV analyses, n = 10 for each genotype at PND5, n = 11 for each genotype at PND9. **(G)** Maximal seizure electroconvulsive threshold (ECT) endpoint (n = 25 WT, 16 HET adults). MWU p< 1x10^-4^. iRMS = root mean square current. PND = postnatal day. Error bars = SEM.

At birth, mutant pups weighed on average 10% less than WT controls (MWU, permuted p = 5x10^-3^) (S5A Fig). This growth impairment was further exacerbated throughout infancy, with PND12 mutants weighing roughly 19% less than controls (MWU permuted p< 0.01 for all time points) (Figs 2A and S5B). This degree of growth impairment persisted throughout the juvenile period into adulthood (MWU permuted p< 0.01 for all time points), and was more evident for female mutants compared to males (S5C–S5E Fig).

Despite weighing significantly less, *Hnrnpu*^+/113DEL^ pups showed a subtle increase in latency to fall at PND6 in the vertical screen test (S5F Fig). Mutants also showed a modest impairment in both righting reflex at PND10 and the 90° negative geotaxis (the time it takes to right 90 degrees from a downward facing position on a wired mesh at a 45° angle) at PND12, highlighting a trend towards delayed sensorimotor function (MWU permuted p = 1x10^-3^ and p = 4x10^-3^, respectively) (S5G and S5H Fig). There was no significant difference in 180° negative geotaxis for any of the time points evaluated (S5I Fig).

To further assess developmental delay in *Hnrnpu*^+/113DEL^ pups, we evaluated separation-induced ultrasonic vocalizations (USVs). USVs are functionally important signals that elicit maternal retrieval and care [36]. Deficits in pup USVs have been reported in various rodent models of neurodevelopmental disease, most notably in models of human communication disorders such as autism and verbal dyspraxia [37–42]. Evaluation of USVs from WT mice revealed the canonical inverted-U shape trajectory from PND3 to PND11, characteristic of normal pups [43] (Fig 2B). The number of WT pup calls increased steadily and peaked at PND7 (Fig 2B). Conversely, *Hnrnpu*^+/113DEL^ pups showed clear deficits in USVs (Fig 2B and 2C), including a striking reduction in the number of calls, particularly at PND5 and PND7 (MWU permuted p = 7x10^3^ and < 1x10^-4^, respectively), with an atypical trajectory characterized by a slow increase in the number of calls that peaked around PND9 (Figs 2B and S5J). Further analysis of USV acoustic properties of mutants at PND5 and PND9 revealed a shorter duration (MWU permuted p = 2x10^-3^ and < 1x10^-4^, respectively) and overall higher frequency (MWU permuted p = 7x10^-3^ and 0.02, respectively) compared to control calls (Fig 2D and 2E). Moreover, mutant vocalizations also trended towards an increased peak amplitude, although this observation was only significant at PND9 (MWU permuted p = 3x10^-3^) (Fig 2F). Overall, these data, combined with growth and milestone studies, are suggestive of global developmental delay in *Hnrnpu*^+/113DEL^ mice.

### *Hnrnpu*^+/113DEL^ adults exhibit seizure susceptibility

Given the presence of early-onset seizures in patients with *HNRNPU* mutations [26–28], we assessed *in vivo* spontaneous and evoked excitability phenotypes using electroencephalography (EEG) and electroconvulsive threshold (ECT) studies, respectively. Despite over 300 total hours of video EEG recordings among *Hnrnpu*^+/113DEL^ adults, there was no evidence of spontaneous generalized epileptiform activity (S6A Fig). Moreover, no spontaneous seizure-like behaviors or sudden death were observed following routine handling of this mouse line at any age.

In epilepsy mouse models, neuronal hyperexcitability can also manifest as greater susceptibility to induced seizure events [44,45]. Evoked seizure susceptibility was therefore measured using electroconvulsive threshold (ECT) studies, where an animal’s seizure response is scored following application of a precise level of electrical stimulus to the brain. The seizure threshold is subsequently determined by averaging the level of stimulus required to elicit a specific behavioral endpoint. *Hnrnpu*^+/113DEL^ mice demonstrated a significantly lower threshold for induction of maximal tonic hindlimb extension seizures, consistent with a greater seizure predisposition (MWU p< 1x10^-4^) (Fig 2G).

Considering the moderate to severe intellectual disability, along with motor and neuromuscular impairments observed in patients with *HNRNPU* mutations [26–28], adult mice were surveyed for exploratory activity, gait, and learning and memory. Results revealed only modest differences between *Hnrnpu*^+/113DEL^ and WT adult mice (S6B–S6P Fig). To further assess cognitive function, an EEG spectral analysis was performed, specifically assessing gamma waves during both wake and sleep. Gamma oscillations are associated with several cognitive functions, such as perceptual and associative learning, object representation, and selective attention [46–49]. Abnormal EEG gamma oscillations have been reported in several neuropsychiatric disorders, including schizophrenia [50], depression [51], and Alzheimer’s disease [52]. *Hnrnpu*^+/113DEL^ animals showed increased gamma oscillations during wake compared to WT animals, yet no difference was observed during slow-wave sleep (S7A–S7D Fig). These results are suggestive of broader cognitive dysfunction in this mouse model.

### Single-cell RNA-sequencing of the neocortex and hippocampus reveals ubiquitous *Hnrnpu* expression

We performed scRNAseq on neocortical and hippocampal samples obtained from *Hnrnpu*^+/113DEL^ and WT littermates at a single postnatal time point. For both brain regions, we evaluated two pups of each genotype, including one of each sex. Cortices and hippocampi were dissected from different mice originating from separate litters. In total, we sequenced 18,171 neocortical cells and 21,487 hippocampal cells.

**Fig 3:**
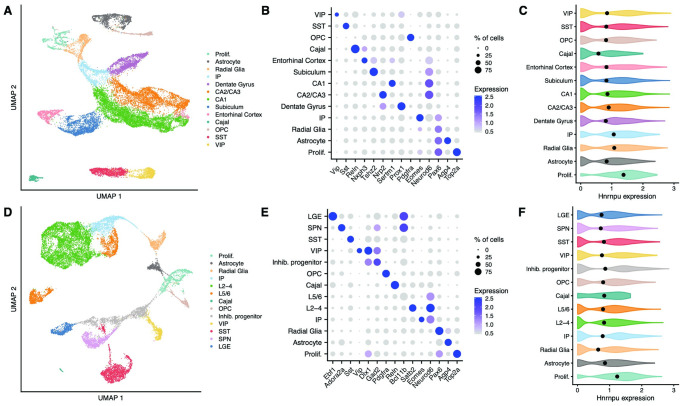
Single-cell RNA-sequencing of wildtype and mutant neocortical and hippocampal cells. **(A)** UMAP plot of all hippocampal cells, colored by cell type. Prolif: proliferative cells; IP: intermediate progenitors; Cajal: Cajal-Retzius cells; OPC: oligodendrocyte precursor cells; SST: SST+ interneurons; VIP: VIP+ interneurons. **(B)** Expression of canonical cell type markers used for annotating each hippocampal cell population. **(C)** Expression of *Hnrnpu* in each population of WT hippocampal cells. **(D)** UMAP representation of all neocortical cells, colored by cell type. L2-4: upper layer (layers 2–4) pyramidal neurons; L5/6 deep layer (layers 5 and 6) pyramidal neurons. **(E)** Expression of canonical cell type markers used for annotating each neocortical cell population. **(F)** Expression of *Hnrnpu* in each population of WT neocortical cells.

Using Seurat [53,54], we harmonized expression data across WT and *Hnrnpu*^+/113DEL^ cells before performing unsupervised clustering (S8A and S8B Fig). We then combined cell clusters into major cell classes based on expression of well-established canonical marker genes (Fig 3B and 3E and S2 Table) [55,56]. In total, we identified 13 distinct cell populations for each brain region (Fig 3A and 3D). Overlapping cell populations included proliferative cells, radial glia, intermediate progenitors, oligodendrocyte precursor cells, and inhibitory subpopulations, including SST and VIP positive interneurons (Fig 3A and 3D). In the neocortex, we identified additional inhibitory neuron clusters, including LGE-derived interneurons, interneuron progenitors, and spinal projection neurons (Fig 3D). We classified neocortical pyramidal neurons into two major populations: upper layers 2 through 4 and deeper layers 5 and 6. We classified hippocampal pyramidal neurons based on their respective hippocampal subfield, including the dentate gyrus, CA1, CA2 and CA3, subiculum, and entorhinal cortex (Fig 3A). *Hnrnpu* was expressed ubiquitously across all neocortical and hippocampal cell populations, though was slightly increased in proliferative cells (i.e. neural stem cells) (Fig 3C and 3F).

### Cell-type-specific differential gene expression analysis

**Fig 4:**
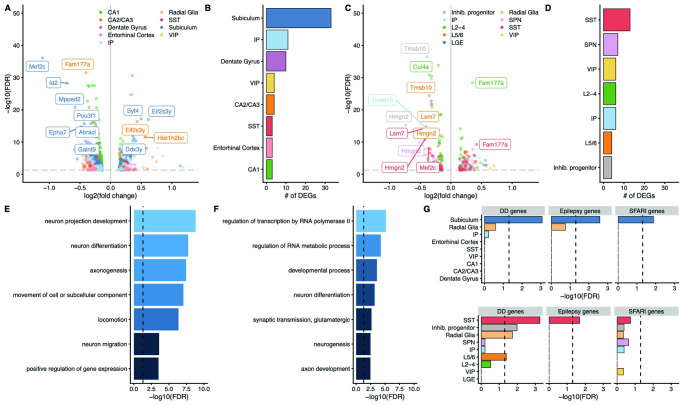
Cell-type-specific dysregulation of gene expression in the hippocampus and neocortex. **(A)** Volcano plot representing differentially expressed genes in the hippocampus. **(B)** Burden of differentially expressed genes in each hippocampal cell type based on downsampled data. **(C)** Volcano plot representing differentially expressed genes in the cortex. **(D)** Burden of differentially expressed genes in neocortical cell types based on downsampled data. **(E, F)** Gene ontology analysis of downregulated genes in the hippocampus and neocortex, respectively. **(G)** Enrichment of developmental delay (DD), epilepsy, and autism (SFARI) genes among nominally significant DEGs in hippocampal (upper panel) and neocortical (lower panel) cell types.

We next performed differential gene expression to identify cell-type-specific perturbations in the *Hnrnpu*^+/113DEL^ brain. We compared gene expression profiles from mutant and WT cells of each population using a linear mixed model (Fig 4A and 4C). In the hippocampus, we detected 955 differential expression events (FDR q< 0.05 and expression change > 10%), composed of 679 unique differentially expressed genes (DEGs) (S3 Table). In the neocortex, we detected 454 differential expression events, composed of 303 unique DEGs (S4 Table). Notably, in the hippocampus there were substantially more downregulated differential expression events (698 genes; 73%) than upregulated (257; 27%). This pattern was not as evident in the neocortex, in which 230 differential expression events resulted in downregulation (51%) compared to 224 that resulted in upregulation (49%). Effect sizes were generally modest. The average absolute log fold change was 0.24 among hippocampal differential expression events and 0.25 among neocortical differential expression events.

### Downregulated genes converge on neuronal processes and pathways

In effort to determine whether DEGs were enriched for certain biological pathways, we performed gene ontology analyses. Downregulated genes in both the mutant hippocampus and neocortex were enriched for several ontologies relevant to the disease phenotype, including neuron projection development, axon guidance, neuron migration, and glutamatergic synaptic signaling (Fig 4E and 4F and S5 Table). Meanwhile, upregulated genes were more strongly enriched for ontologies relevant to cellular growth, differentiation, protein translation and localization (S5 Table).

### An increased burden of DEGs in excitatory neurons of the mutant subiculum

We next tested whether certain cell types are more vulnerable to the reduced expression of *Hnrnpu* than others. First, we downsampled the data to compare the same number of cells across all cell types (n = 300 cells per cell type; S9A and S9B Fig). We re-ran the differential gene expression analysis on these downsampled data and found that excitatory neurons derived from the subiculum showed the highest burden of DEGs across all cortical and hippocampal cell types (Fig 4B and 4D). The next strongest burden was identified in SST+ interneurons of the neocortex. Interestingly, the neocortical SST+ interneurons had more DEGs than hippocampal SST+ interneurons.

Because patients with *HNRNPU* haploinsufficiency often have autistic features, developmental delay, and seizures, we next tested for the enrichment of genes associated with these conditions among each population of cells. Here, we relaxed the significance threshold for DEGs to an FDR < 0.1 and expression change of at least 5% given the relatively small sizes of these gene sets and weak expression of these disease genes at PND0. Strikingly, we observed an overrepresentation of developmental delay, epilepsy, and autism genes among the downregulated genes in the subiculum-derived pyramidal cells (Fig 4G). No other cell type showed this strong of an enrichment for all three disease gene sets. We also observed an enrichment of developmental delay and epilepsy genes among downregulated genes in SST+ neocortical interneurons (Fig 4G). Altogether, these results point to the subiculum as a potentially vulnerable region that may play an important role in mediating the pathophysiology underlying *HNRNPU-*DEE.

### *Mef2c* is the most downregulated gene in the mutant subiculum

**Fig 5:**
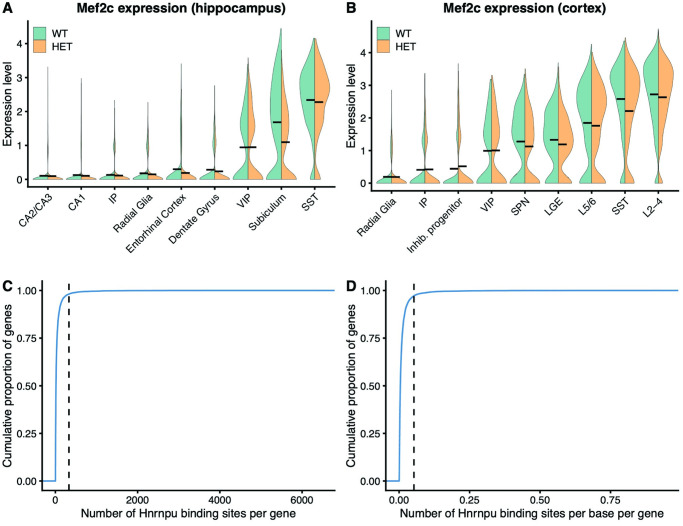
Dysregulation of *Mef2c*. **(A, B)** Expression of *Mef2c* in wildtype and mutant hippocampal and cortical cells. Black lines represent median expression levels in each genotype. **(C)** Cumulative distribution plot representing Hnrnpu binding sites per gene, derived from CLIP-seq data performed on cardiac tissue. Dotted line represents the number of Hnrnpu binding sites in *Mef2c*. **(D)** Same as (C), except that the number of binding sites is normalized by transcript length.

The most downregulated gene observed in the subiculum was *Mef2c*, which showed a roughly 50% decrease in expression (log_2_ fold change = -1.11, FDR = 8 x 10^−37^) (Fig 5A). This effect size was among the largest observed fold-changes of genes differentially expressed in both the neocortex and hippocampus. Expression of *Mef2c* in the hippocampus was primarily confined to both subiculum-derived pyramidal neurons and SST+ interneurons (Fig 5A). Its expression was more widespread in the cortex, including expression in upper and deep layer pyramidal neurons, along with SST+ and VIP+ interneurons (Fig 5B). Interestingly, despite this widespread expression, the only other cells in which *Mef2c* was significantly downregulated were SST+ interneurons, yet to a lesser degree than subiculum-derived pyramidal neurons (FDR q = 9 x 10^−9^; log_2_ fold-change = -0.36) (Fig 5A and 5B). This finding further highlights the presence of cell-type-specific effects upon the loss of ubiquitously expressed hnRNP U protein.

To assess whether hnRNP U likely functions to directly regulate the expression of *Mef2c*, we examined the number of hnRNP U binding sites for each gene expressed in the brain of mice. Using available hnRNP U CLIP-sequencing data derived from mouse cardiac tissue, we found that *Mef2c* contains more hnRNP U binding sites than 99% of the other genes assessed (Fig 5C). This effect persisted when we normalized the number of binding sites by gene length (Fig 5D). Together, these results suggest that *Hnrnpu* plays an important role in regulating expression of *Mef2c* at this timepoint.

### Candidate compounds for transcriptomic reversal of the *Hnrnpu*^*+/113DEL*^ disease expression signature

Because heterozygous loss of *Hnrnpu* leads to widespread cell-type-specific dysregulation of gene expression, identifying targeted therapeutics could prove especially challenging. However, transcriptomic signature reversal—a paradigm well-developed in cancer—may provide one particularly promising avenue for drug discovery for both this disease and other neurodevelopmental diseases caused by genes that directly influence the transcriptome. This paradigm posits that if gene expression changes underlie the pathophysiology of a particular disease, then correction of this transcriptomic signature toward a normal state may have therapeutic potential. Transcriptomic reversal requires the comparison of a disease gene expression signature and the gene expression signatures of cells treated with small molecules. Small molecules that elicit expression changes most anticorrelated with the disease signature are prioritized for further validation. The Connectivity Map (CMAP) [57,58] provides publicly available expression signatures derived from cancer cell lines treated with thousands of small molecules.

Transcriptomic reversal approaches that have leveraged the CMAP and other resources have not only successfully identified targeted therapeutics in cancer [7,59,60], but for other diseases too, including diabetes and inflammatory bowel disease [8,61]. However, this approach has not been successfully applied to transcriptome-mediated neurodevelopmental conditions. Given the pleiotropic, cell-type-specific effects of *Hnrnpu* haploinsufficiency in disease-relevant brain regions, we expect that this approach will require scRNAseq-derived signatures.

To examine the importance of cell-type-specific effects, we compared compounds predicted to reverse the subiculum gene expression signature versus compounds predicted to reverse a “pseudo-bulk” hippocampal signature (i.e. the average gene expression changes across all cell types). We specifically focused on the downregulated genes in the disease signatures, as these genes were enriched for biologically relevant pathways and disease genes. The CMAP uses a “connectivity score” to assess each compound’s ability to reverse the query signature [57]. This score ranges from -100 to +100, with a score of -100 indicating complete reversal.

For the subiculum-derived signature, 128 compounds received a Connectivity Score less than the CMAP’s recommended cutoff of -90 (S10A Fig and S6 Table). 98 compounds received a score below this threshold for the pseudo-bulk derived signature (S10B Fig and S6 Table). Only 40 (31%) of these candidate compounds overlapped between the two queries (S10E Fig). Furthermore, among the top 20 compounds prioritized per signature, only four compounds overlapped (linifanib, Merck60, etinostat, and BMS-345541) (S10C and S10D Fig). The classes of compounds among these 20 compounds prioritized for each signature were also different. An overwhelming majority of pseudo-bulk prioritized compounds were HDAC inhibitors (70%), whereas the most common drug class for the subiculum-prioritized signatures were tubulin inhibitors and microtubule stabilizing agents (25%).

Therefore, if transcriptional dysregulation of a specific cell type, such as excitatory neurons in the subiculum, contributes substantially to the pathophysiology underlying *HNRNPU* haploinsufficiency, bulk RNA-sequencing may not generate adequate signatures to identify the compounds most likely to target the relevant disease mechanisms. Experimental validation will be required to verify that these candidate compounds in fact reverse the transcriptomic signatures and rescue Hnrnpu disease phenotypes. Nonetheless, these results highlight the potential importance of deriving cell-type-specific disease expression signatures for transcriptomic signature reversal approaches.

## Discussion

Identifying and modeling germline mutations associated with developmental and epileptic encephalopathies provides the unique opportunity to develop targeted therapeutics [62]. While most of these mutations tend to occur in genes that encode ion channels or synaptic transmission proteins, a subset of these genes can be thought of as causing disease through their effects on the transcriptome [63,64]. Unfortunately, elucidating disease mechanisms for this subset of genes is often difficult, as the encoded proteins regulate the expression of thousands of target genes. To address this problem, using our precision genetic mouse model, we evaluated the potential utility of a transcriptome-guided precision medicine approach for hnRNP U-mediated neurodevelopmental disease, relying on brain-region and single cell-level gene expression profiles to highlight vulnerable cell types and key dysregulated genes.

### Near wildtype expression of *HNRNPU* is necessary for normal development

Intriguingly, like the human cortical organoid models previously described [34], we observed that loss of a single copy of mouse *Hnrnpu* does not lead to 50% reduction in gene expression as predicted by the protein truncating nature of the frameshift mutation. Despite only this partial reduction in expression, heterozygous intercrosses failed to produce any homozygous progeny, consistent with embryonic lethality in mice. These findings are also consistent with the observation that mutant ES cells containing embryonically lethal homozygous hypomorphic *Hnrnpu* mutations showed only a 40–80% decrease in *Hnrnpu* transcript levels [31].

While the mechanism underlying this partial loss of *Hnrnpu* expression remains unknown, potential explanations include the enhanced stability of *Hnrnpu* transcripts, such as through autoregulation via self 3’UTR binding [20] or the compensatory upregulation of *Hnrnpu* expression through mechanisms like transcriptional adaptation [65]. Differentiating between these possibilities could unveil future avenues for molecular therapy, such as targeting the transcriptional adaptation mechanism to further upregulate *Hnrnpu* expression.

### *Hnrnpu*^+/113DEL^ mice model aspects of *HNRNPU* developmental and epileptic encephalopathy

Here we performed a comprehensive neurophysiological characterization of an *HNRNPU* neurodevelopmental disease model. *Hnrnpu*^+/113DEL^ mice displayed several phenotypes that overlap in presentation with the human disorder [26–28]. Mutant mice demonstrated global developmental delay as evidenced by significantly impaired growth, delayed sensorimotor function and striking deficits in separation-induced pup ultrasonic vocalizations. The short and high-pitched presentation of mutant calls are likely less effective as communicative signals [66,67], and may contribute to the exacerbation of the physical growth impairment noted during the perinatal period from poor maternal care. In adulthood, mutant mice also exhibited greater seizure susceptibility and increased gamma oscillations while awake, suggestive of broader cognitive dysfunction. These findings support the utility of this model to further investigate and interrogate the molecular underpinnings of *HNRNPU*-mediated disease phenotypes. Our study also builds upon prior behavioral work conducted in an independent heterozygous *Hnrnpu* knockout model that demonstrated altered circadian-mediated locomotor and metabolic activity in mice carrying an out-of-frame deletion of exons 3 through 6 [68].

### *Hnrnpu* is important for sustaining expression of neuronally expressed genes

Through single-cell RNA-sequencing of the neocortex and hippocampus, we identified widespread transcriptional dysregulation across all the neuronal cell types examined in mutant mice. The overall magnitude of these expression changes was generally modest—an observation similar to that described in Rett Syndrome, both in post-mortem human tissue and *Mecp2* mouse models, as well as a mouse model of CHD8-mediated neurodevelopmental syndrome [69–71]. Recent findings from a cross-species comparison suggest that the extent of transcriptional dysregulation is largely temporally-dependent, with embryonic time points showing a greater degree of perturbance that was anticorrelated to expression changes observed in the perinatal period [34]. In the current study, both the neocortex and hippocampus showed downregulation of genes that were particularly enriched for important, disease-associated ontologies, including neuronal migration and axon guidance pathways. These findings are in line with the *in vivo* neurological phenotype of mutant mice, and allude to an important role for hnRNP U in regulating the expression of many disease-relevant, neuronally expressed genes.

### Cell-type-specific effects upon heterozygous loss of *Hnrnpu*

Interestingly, at this perinatal time point, we found that the greatest burden of gene expression dysregulation upon heterozygous loss of *Hnrnpu* occurred in pyramidal cells of the subiculum. Downregulated genes in this cell type were also enriched for known neurodevelopmental disease genes including those associated with autism, developmental delay, and epilepsy. Although the subiculum functions as the primary output of the hippocampus and is important for normal hippocampal-related functions such as learning and memory and stress response, this brain region has also been implicated in pathological conditions such as the generation and spread of temporal lobe seizures [72–76]. While the location of seizure onset was not reported for most individuals with *HNRNPU* mutations, two patients reportedly had temporal lobe epilepsy [26,28]. Furthermore, haploinsufficiency of the most downregulated gene in the subiculum, *Mef2c*, is associated with a neurodevelopmental disorder showing significant phenotypic overlap to patients with *HNRNPU* mutations [77,78]. While these findings only reflect a single snapshot of time and do not account for other transcriptional insults that may have occurred during neurodevelopment, these results do demonstrate relevant cell-type and brain-region specific gene expression changes that may contribute to the general dysfunction and subsequent pathological phenotypes.

### Transcriptomic reversal of mutant cell-type-specific signatures as a therapeutic approach

Widespread and cell-type-specific transcriptomic defects present a challenge regarding pinpointing targeted therapeutics. However, transcriptomic signature reversal may provide one route forward. This approach has been successful in identifying compounds that ameliorate seizures in non-genetic models of epilepsy [79]. Through querying the Connectivity Map [57,58], we find numerous compounds that may reverse disease gene expression signatures. We find that the prioritized compounds, however, are sensitive to the use of cell-type-specific versus bulk-derived expression signatures. Therefore, in addition to considering temporal changes in gene expression, identifying the most vulnerable cell types for each transcriptome-mediated epilepsy gene may be another crucial factor for the successful application of this approach in neurodevelopmental diseases.

## Conclusion

Here we characterized the neurophysiological and cell-type-specific transcriptomic effects of a mouse model of *HNRNPU* developmental and epileptic encephalopathy. This model system demonstrated developmental impairment, seizure susceptibility and cognitive dysfunction, recapitulating aspects of the human condition. We further observed pervasive gene expression changes of modest effect that occurred in a cell-type-specific manner, including many neuronally-expressed genes that converged on important neuronal pathways. This was accompanied by a nearly 50% reduction in expression of a single neurodevelopmental disease gene, *Mef2c*, in pyramidal neurons of the subiculum—a cell-type particularly vulnerable to heterozygous loss of *Hnrnpu*. Overall, these findings imply a complex role for *Hnrnpu* in gene expression regulation of the brain—a notion supported by its multifaceted role in regulating multiple levels of gene expression. This complexity is likely further amplified by many factors including context-dependent interactions with other gene expression regulators, developmental timing, sex-specific effects and species-specific variation that were not examined as part of this work, but do warrant further consideration. These results also support the exploration of alternative therapeutic approaches, with transcriptomic signature reversal of key, vulnerable cell-types as a promising, novel strategy for transcriptome-mediated neurodevelopmental disease.

## Materials and methods

### Ethics statement

All animal protocols were approved by the Institutional Animal Care and Use Committee (IACUC) at Columbia University Irving Medical Center.

### Mouse husbandry

*Hnrnpu*^+/113DEL^ mice were generated through The Jackson Laboratory’s Genome Engineering Technology core using a CRISPR-Cas9 strategy targeting exon 1 of mouse *Hnrnpu*. Of 15 founder mice that survived, 6 appeared most promising based on TIDE analysis and were further evaluated using TOPO-TA cloning to validate the corresponding mutant alleles. A founder containing an out-of-frame 113-bp deletion was further expanded and maintained on a C57BL/6NJ background. All experiments were performed on the inbred background except for ECT studies, which were performed on the F1 hybrid background C57BL/6NJ (005304 JAX stock) x FVB/NJ (001800 JAX stock), as mutants on an inbred C57BL/6NJ were significantly smaller than WT controls. WT littermates were used as controls in all experiments. All mice were maintained in ventilated cages with controlled humidity at ~60%, 12h:12h light:dark cycles (lights on at 7:00AM, off 7:00PM) and controlled temperature of 22–23°C. Mice had access to regular chow and water, ad libitum. Breeding cages were fed a high fat breeder chow. Mice were maintained and all procedures were performed within the Columbia University Institute of Comparative Medicine, which is fully accredited by the Association for Assessment and Accreditation of Laboratory Animal Care.

### Genotyping

DNA was extracted from tail or ear clippings using the Kapa Mouse Genotyping Standard kit (KAPA Biosystems) and stored at -20°C. PCR was performed with 2x MyTaq HS Mix (Bioline), using the following Hnrnpu primers: FWD = 5’-GTCCGTTCTGCAGCAGCACT-3’, REV = 5’- TTACCTCCCGCCTGCTGTTG-3’. This amplifies a 745-bp product from the WT allele and a 632-bp product from the mutant allele.

### Primary neuronal culture

P0 pups were tail sampled, weighed and genotyped for the *Hnrnpu* 113-bp deletion. Mutant and wildtype pups were decapitated, and cortex and hippocampus were separately dissected in cold Hibernate A (Thermo Fisher). Tissue was diced into smaller pieces and dissociated in a solution containing pre-warmed Hibernate A, papain, and DNase for 20 min at 37°C. Dissociated tissue was then centrifuged at 300 g for 5 min at room temperature (RT), resuspended in pre-warmed Hibernate A, and triturated to further dissociate. Undissociated tissue was allowed to settle to the bottom of the tube, and the single cell suspension was transferred to a new tube and centrifuged at 300 g for 5 min at RT. The cell pellet was resuspended in complete medium containing Neurobasal A (Thermo Fisher), B27 Plus (Thermo Fisher), 1% FBS, Hepes, Glutamax and Penn/Strep. Cell viability and counts were obtained using a trypan blue exclusion assay, then further resuspended to the desired cell concentration using complete medium supplemented with laminin (5 ug/ml). Both cortical and hippocampal cells were plated on PDL-coated 12 mm coverslips in a 24-well dish at a density of 200,000 cells. Complete medium was changed the following morning to Neurobasal A, B27 Plus, Hepes, Glutamax and Penn/Strep, and 50% medium changes were subsequently performed every other day.

### Immunocytochemistry

On day in vitro 9 (DIV9), mouse primary cortical and hippocampal cells were washed 2x with 1X PBS, fixed for 15 min in 4% paraformaldehyde at RT, and again washed 2x with 1X PBS. Cells were incubated in a staining solution comprised of 5% donkey serum, 1% BSA, 0.3% TritonX-100 in 1X PBS for 15 min at RT, then subsequently incubated in the primary antibody diluted in the staining solution for 2 hr. at RT. Cells were washed 4x with 1X PBS, 0.2% TritonX-100, incubated with the fluorophore conjugated secondary antibody in staining solution for 30 min at RT then washed 4x with 1X PBS, 0.2% TritonX-100. Coverslips were mounted using ProLong-Antifade with DAPI on Superfrost Plus Microscope slides and allowed to dry in the dark prior to imaging. Imaging was performed using the Zeiss Axio Observer.Z1 Fluorescence Motorized Microscope and associated Zen2 Pro imaging software. Downstream image processing was performed using Adobe Photoshop, using auto-brightness and contrast for each individual channel and merged image. Primary antibodies used include: Mouse anti-Map2 at 1:500 (Sigma M4403), mouse anti-GFAP at 1:100 (Abcam ab10062), mouse anti-Gad67 at 1:500 (Millipore MAB5406), mouse anti-Satb2 at 1:100 (Abcam ab51502), rat anti-Ctip2 at 1:250 (Abcam ab18465), rabbit anti-HNRNPU at 1:500 (Abcam 20666). Secondary antibodies include: 488 and 568 Alexa Fluorophore conjugated donkey anti-mouse, donkey anti-rabbit and donkey anti-rat (Invitrogen), at 1:1000 dilution.

### Western blotting

Dissected tissue was snap frozen in liquid nitrogen and stored at -80°C until time of extraction. Tissue was thawed on ice and homogenized using a motorized pestle in RIPA buffer containing both protease and phosphatase inhibitor cocktails (Roche). Lysis was completed for 15 minutes on ice. Samples were subsequently centrifuged at full speed for 20 minutes at 4°C. The resulting supernatant was collected, and protein was quantified using the BCA method (Pierce) with BSA as a standard. All western blots were performed using the Novex NuPAGE system (Invitrogen). Protein lysates were diluted in LDS sample buffer and reducing agent, and heated at 70°C for 10 min. Using the Xcell SureLock Mini Cell gel box, a total of 5 ug of reduced protein lysates were loaded onto a 4–12% gradient Bis-Tris gel in 1X SDS Running buffer (Invitrogen) supplemented with the NuPAGE antioxidant, and ran at 180 V for 1–1.5 hrs. Using the Xcell II Blot Module, proteins were subsequently transferred to a 0.2 um methanol activated PVDF membrane at 30 V for 1.25 hrs at 4°C in Transfer buffer containing 20% methanol. Membranes were blocked for 1 hr at RT in 5% milk, then incubated overnight in the hnRNP U primary antibody at 1:1000 (Rabbit polyclonal against C-terminus: Abcam ab20666; Rabbit monoclonal against N-terminus: Abcam ab180952) diluted in 5% BSA. Blots were washed 3x for 10 min in PBST, incubated at RT for 1 hr in a secondary HRP-conjugated anti-rabbit (at 1:10,000) diluted in 5% BSA, then further washed 3x for 10 min in PBST. Proteins were incubated for 5 min in a standard ECL substrate (Pierce) and developed with either a Kodak X-OMAT 2000A Processor or iBright FL1000 Imaging system (Invitrogen). For a loading control, blots were subsequently incubated in an HRP-conjugated b-Actin secondary at 1:1000 (Santa Cruz #sc-47778) diluted in 5% BSA for 1 hr at RT, then washed and developed as previously described. Densitometry analysis was performed using the iBright FL1000 imager. Specifically, the Local Background Corrected Density, LBCD, (background-corrected volume/area) of each hnRNP U-probed sample was first normalized to the corresponding LBCD of β-Actin to generate an hnRNP U/β-Actin ratio. WT and HET ratios were further divided by the average WT hnRNP U/β-Actin ratio and plotted individually.

### qRT-PCR

Mouse cerebral cortex: Cortical tissue was collected and immediately stored in RNALater Stabilization Solution (Qiagen) at 4°C. After 24 hrs., the RNALater was subsequently removed, and samples were stored long term at -80°C. For RNA extraction, tissue was first mechanically homogenized using a motorized pestle in RLT buffer (Qiagen) supplemented with b-mercaptoethanol, then further homogenized using a QIAshredder spin column (Qiagen). RNA was extracted using the RNeasy Mini kit (Qiagen) per protocol instructions, and the resulting RNA concentration and purity were assessed using a NanoDrop. A total of 2.5 ug of RNA was used for the reverse transcription reaction, which was performed using the SuperScript IV First-Strand Synthesis System (ThermoFisher) with random hexamer priming. Resulting cDNA was used as input into pre-validated TaqMan Gene Expression Assays (ThermoFisher) and run with the TaqMan Fast Universal PCR MasterMix 2x (Applied Biosystems). The following TaqMan probes were purchased from ThermoFisher: mHnrnpu Mm00469329_m1 (spans exons 1–2) and mCyc1 Mm00470540_m1 (spans exons 1–2). A total of six biological replicates were evaluated. TaqMan assays were run on a QuantStudio 5 Real-Time PCR System (Applied Biosystems) using the comparative Ct method.

All qRT-PCR analyses were performed using QuantStudio Design and Analysis Software v1.2 and Microsoft Excel. To analyze for gene expression differences, raw Ct values were first averaged across technical replicates, which ranged from 2–4 for each sample. For samples with a Ct standard deviation >0.3, a technical replicate was filtered out if its Ct value was one standard deviation above or below that of the mean of the replicates. We required samples to have at least technical duplicates to be considered in the final analysis. Delta Ct values were determined by calculating the difference between mean Ct values for experimental samples and corresponding loading controls. Delta delta Ct values for both HET and WT samples were then calculated using the average WT delta Ct from the corresponding time point and experimental batch, and then were independently plotted along with SEM. Statistical analyses were performed using Welch’s two-sample t-test.

### Morphological studies

Brains were extracted from PND0 pups and fixed in Bouin’s solution overnight at room temperature. Fixed brains were embedded in paraffin with service provided by Columbia University’s Molecular Pathology Core Facility. Coronal sections in 5 μm thickness were obtained using a microtome (Leica RM2125RT) and subjected to hematoxylin and eosin (H&E) staining. Briefly, the slices were deparaffinized in xylene and rehydrated with ethanol and water. Slices were then stained in hematoxylin, counterstained with eosin and subsequently dehydrated with ethanol and xylene. Stained slices were mounted with coverslips using Permount (Fisher Chemical). Images were acquired using a Nikon Eclipse E800 Microscope packaged with NIS-Elements DV.4.51.00 imaging software. For presentation purpose, full size images were subjected to automatic brightness and contrast adjustment using Adobe Photoshop. Brain measurements were collected using ImageJ. The measurements were normalized to respective pup body weight. Student’s T-test was performed on each set of measurements and Bonferroni corrected for multiple comparisons.

### Pup developmental milestones

On PND2, pups were tattooed on the bottom of their paws for identification using a 25–30 G needle and Ketchum tattoo ink, following the AIMS tattoo chart. The following developmental milestone studies were completed on P4, P6, P8, P10 and P12 within a 3 min time window for each test subject.

Righting reflex: the latency to flip over from supine to prone position on all fours. Pups were gently placed on their backs on a hard surface and released. A stopwatch was used to measure the total time for each pup to right itself. The cutoff latency was 30 s.

Negative geotaxis: the latency to face upwards from a downward-facing start position on an inclined mesh screen. Pups were placed at a downward facing position on a 45° inclined screen and released. A stopwatch was used to record the time it takes for each pup to turn 90° then 180°. The cutoff latency was 30 s. If the pup failed the trial by falling while turning, they were scored the maximum cutoff latency time of 30 s.

Vertical screen: the latency to fall from a vertically positioned wire mesh screen. Pups were positioned to grasp the screen. Using a stopwatch, the time until fall was recorded. The minimum and maximum latency allowed was 1 s and 30 s, respectively.

For each developmental test, every pup underwent two successive trials that were subsequently averaged and used in the downstream analyses. Throughout the testing period, the experimenter was blinded to genotype. The mean and standard error of the mean (SEM) for both genotypes were plotted across all tested timepoints. Permuted MWU p-values were reported for each time point. Briefly, WT and HET data were pooled, randomly sampled and MWU p-values calculated 10,000 times. Permuted MWU p-values were calculated as the total number of randomly sampled MWU p-values that fell below the actual MWU p-value divided by the total number of permutations (10,000).

### Pup ultrasonic vocalizations (USVs)

USVs were assessed on P3, P5, P7, P9 and P11. Each pup was gently removed from the nest and placed in a small, plastic container containing a 0.5 cm layer of fresh bedding. The cage lid was immediately returned to avoid irritating the dam and remaining pups in the nest. The container holding the pup was placed immediately into a sound-attenuating environmental chamber (Med Associates, St. Albans, VT, USA). After a 3 min recording, each pup was marked and returned to the nest. Ultrasonic vocalizations were recorded with an Ultrasound Microphone (Avisoft UltraSoundGate condenser microphone capsule CM16, Avisoft Bioacoustics, Berlin, Germany) sensitive to frequencies of 10–180 kHz and using the Avisoft Recorder (Version 4.2) software. Sampling rate was 250 kHz, format 16 bit. Ultrasonic vocalizations were analyzed using Avisoft SASLab Pro software (Avisoft Bioacoustics). Spectrograms were generated for each 1 min audio file, with an FFT-length of 512 points and a time window overlap of 75% (100% Frame, Hamming window). The spectrogram was generated at a frequency resolution of 488 Hz and a time resolution of 1 ms. A lower cut-off frequency of 15 kHz was used to reduce background noise outside the relevant frequency band to 0 dB. Calls were inspected visually and manually labelled. Summary statistics were generated by Avisoft SASLab Pro and analyzed using Prism. All calls emitted over the 3 min recordings were quantified. For the qualitative analysis, given the extensive manual review required to measure duration, peak frequency, and amplitude, we chose to limit this analysis to 2 time points, 1 near the beginning (P5) and the other near the end (P7) of the canonical inverted U-shaped curve. One 1 min file (out of three 1 min files) that included the most USVs were analyzed for each mouse. Mean and SEM were plotted, and permuted MWU p-values were reported for each time point as described for pup developmental milestone tests.

### Electroencephalography (EEG)

Video EEGs were performed on 6- to 8-week-old adult mice. Mice were anesthetized with tribromoethanol (250 mg/kg delivered via intraperitoneal injection, Sigma Aldrich cat# T48402), and three small burr holes were drilled through the skull 2 mm lateral to the midline (1mm rostral to the bregma on both sides and 2mm caudal to the bregma on the left). One hole was also drilled over the cerebellum as a reference. Four Teflon-coated silver wires soldered onto pins of a microconnector (Mouser electronics cat# 575–501101) were placed in between the dura and brain. A dental cap was applied on top. Each mouse was provided the post- operative analgesic Carprofen (5 mg/kg subcutaneous Rimadyl) and allowed a recovery period of at least 48 hours prior to recording. Signal was obtained on either a Grael II EEG amplifier (Compumedics) or Natus Quantum amplifier (Natus Neuro). Data was analyzed with either Profusion 5 (Compumedics) or NeuroWorks (Natus Neuro). Differential amplification recordings were recorded pairwise between all three electrodes and the reference, resulting in 6 total channels for each subject. Mouse behavior was captured throughout the recording period through video using a Sony IPELA EP550 camera with infrared light for dark recordings. Each mouse was recorded for 24–48 hrs. continuously.

EEG spectral analysis was carried out using fast Fourier transform (FFT) over a 5 s sliding window, sequentially shifted by 2 s increments (bins). Brain states were semi-automatically classified into wake and slow-wave sleep (SWS, or NREM sleep) using a custom-written MATLAB program (wake: desynchronized EEG; SWS: synchronized EEG with high-amplitude, delta frequency (0.5–4 Hz) activity). Semi-automated classification was validated manually by trained experimenters. Note that REM sleep was not classified in this study due to lack of EMG signals. EEG power was normalized to the total power in each mouse. Then, low gamma power (30-50Hz) in different brain states across the entire 24-h recording session was calculated and compared between the mutant and wildtype animals.

### Electroconvulsive threshold (ECT) studies

All tests were performed on 6- to 8-week-old mice. Transcorneal electrodes were used to deliver a predefined stimulus with the Ugo Basile Model 7801 electroconvulsive device. High frequency (HF) electroshock was performed with the following fixed settings: 1.6 ms pulse width, 0.2 s shock duration and 299 Hz pulse frequency with variable settings of 4–12 mA amplitude. The individual threshold for each mouse was determined by testing in 0.5 mA intervals on sequential days until the threshold was reached. The behavioral endpoint evaluated was a maximal tonic hindlimb extension seizure, which often start with tonic extension of the forelimbs that evolves into full tonic hindlimb extension. The overall stimulus is calculated as the iRMS (integrated root mean square, or the integrated area under the curve) using the following equation: sq. root frequency (Hz) x pulse width (ms) x duration (s) x amplitude (mA).

### Adult behavioral tests

Elevated Plus Maze test (EPM): This classic test for anxiety-like behavior is based on rodents’ innate fear for height and open space. The elevated plus-maze consists of two open arms (30 cm x 5 cm) and two closed arms (30 x 5 x 15 cm) extending from a central area (5 x 5 cm). Photo beams embedded at arm entrances register movements. Room illumination was approximately 5 lux. The test begins by placing the subject mouse in the center, facing a closed arm. The mouse is allowed to freely explore the maze for 5 min. Time spent in the open arms and closed arms, the junction, and number of entries into the open arms and closed arms, are automatically scored by the MED-PC V 64bit Software (Med Associates). At the end of the test, the mouse is gently removed from the maze and returned to its home cage. The maze is cleaned with 70% ethanol and wiped dry between subjects.

Open Field exploratory activity: The open field test is the most used general test for locomotor activity. Each mouse is gently placed in the center of a clear Plexiglass arena (27.31 x 27.31 x 20.32 cm, Med Associates ENV-510) lit with dim light (~5 lux), and is allowed to ambulate freely for 60 min. Infrared (IR) beams embedded along the X, Y, Z axes of the arena automatically track distance moved, horizontal movement, vertical movement, stereotypies, and time spent in center zone. At the end of the test, the mouse is returned to the home cage and the arena is cleaned with 70% ethanol followed by water and wiped dry.

Catwalk: Free-pace walking was evaluated using the Catwalk XT system (Noldus Information Technology) which consists of an illuminated walled glass walkway (130 cm x 10 cm) and a high-speed camera underneath. Light is reflected and illuminates the stimulus (footprint) when downward pressure is applied. Walking patterns are captured with a high-speed camera mounted underneath the walkway. The experiment was done with dim room illumination (30 lux). The mouse is allowed to traverse the walkway as many times as needed to obtain at least 3 compliant runs (runs with a speed variation under 80% in 20 seconds or less). Pilot experiments using a 60% speed variation limit (most common in the literature) proved to be too stringent for most heterozygous mice. Parameters automatically collected by the software include, but are not limited to, paw statistics, intensity measures, stride length, width, base of support, distance between ipsilateral prints, cadence, % limb support, regularity index, speed, and speed variation. A highly trained experimenter visually inspected all automatically scored runs, and manually classified any prints that were too ambiguous for the software to identify accurately. The walkway is cleaned with paper towel moistened with 70% ethanol and wiped dry between trials.

Acoustic startle response: Acoustic startle response was tested using the SR-Laboratory System (San Diego Instruments, San Diego, CA). Test sessions began by placing the mouse in the Plexiglass holding cylinder for a 5-min acclimation period. For the next 8 min, mice were presented with each of six trial types across six discrete blocks of trials, for a total of 36 trials. The inter-trial interval was 10–20 s. One trial type measured the response to no stimulus (baseline movement). The other five trial types measured startle responses to 40 ms sound bursts of 80, 90, 100, 110 or 120 dB. The six trial types were presented in pseudorandom order such that each trial type was presented once within a block of six trials. Startle amplitude was measured every 1 ms over a 65 ms period beginning at the onset of the startle stimulus. The maximum startle amplitude over this sampling period was taken as the dependent variable. Background noise level of 70 dB was maintained over the duration of the test session.

Fear Conditioning: This is a classic test for conditioned learning. Training and conditioning tests are conducted in two identical chambers (Med Associates, E. Fairfield, VT) that were calibrated to deliver identical foot shocks. Each chamber was 30 cm × 24 cm × 21 cm with a clear polycarbonate front wall, two stainless side walls, and a white opaque back wall. The bottom of the chamber consisted of a removable grid floor with a waste pan underneath. When placed in the chamber, the grid floor connected with a circuit board for delivery of scrambled electric shock. Each conditioning chamber is placed inside a sound-attenuating environmental chamber (Med Associates). A camera mounted on the front door of the environmental chamber recorded test sessions which were later scored automatically, using the VideoFreeze software (Med Associates, E. Fairfield, VT). For the training session, each chamber is illuminated with a white house light. An olfactory cue is added by dabbing a drop of imitation almond flavoring solution (1:100 dilution in water) on the metal tray beneath the grid floor. The mouse is placed in the test chamber and allowed to explore freely for 2 min. A pure tone (5 kHz, 90 dB) which serves as the conditioned stimulus (CS) is played for 30 s. During the last 2 s of the tone, a foot shock (0.5 mA) is delivered as the unconditioned stimulus (US). Each mouse received three CS-US pairings, separated by 90 s intervals. After the last CS-US pairing, the mouse is left in the chamber for another 120 s, during which freezing behavior is scored by the Video Freeze software. The mouse is then returned to its home cage. Contextual conditioning is tested 24 h later in the same chamber, with the same illumination and olfactory cue present but without foot shock. Each mouse is placed in the chamber for 5 min, in the absence of CS and US, during which freezing is scored. The mouse is then returned to its home cage. Cued conditioning is conducted 48 h after training. Contextual cues are altered by covering the grid floor with a smooth white plastic sheet, inserting a piece of black plastic sheet bent to form a vaulted ceiling, using near infrared light instead of white light, and dabbing vanilla instead of banana odor on the floor. The session consisted of a 3 min free exploration period followed by 3 min of the identical CS tone (5 kHz, 90 dB). Freezing is scored during both 3 min segments. The mouse was then returned to its home cage. The chamber is thoroughly cleaned of odors between sessions, using 70% ethanol and water.

Y maze: The Y-maze is a standard test for assessing short term memory in mice, based on the mouse’s natural tendency to explore novel locations. Memory impairment is indicated by failing to spend more time exploring the novel arm than the familiar arms. The test is conducted in the Y maze (Maze Engineer) consisting of three arms of equal arm lengths (35 cm), arm lane width (5 cm), wall height (10 cm). One arm is the start arm, with a “=“ sticker velcroed on the wall, to the end of the arm. The two stickers (bus and plane) are velcroed on the wall at the end of the other two arms. The placement of the stickers was counterbalanced across animals. The novel arm preference test consists of two trials. In trial 1, each mouse is placed in the designated start arm and allowed access the start arm and the one other arm for 10 min. The third arm is blocked with an opaque door. At the conclusion of trial 1, the mouse was placed in a temporary holding cage for 10 min. For trial 2, the subject mouse was returned to the start location, and allowed to explore all arms for 5 min. A camera mounted above the maze and interfaced with the Ethovision software (Noldus Information Technology) automatically records distance traveled, arm entries, and time spent in each arm. The maze was cleaned with 50% ethanol and allowed to dry between trials and between animals.

Morris water maze: Spatial learning and reversal learning were assessed in the Morris water maze using procedures and equipment as previously described [80]. The apparatus was a circular pool (120 cm diameter) filled 45 cm deep with tap water rendered opaque with the addition of non-toxic white paint (Crayola, Easton, PA). Distal room cues were door, chairs, computers, and proximal cues are two 20cm x 20cm stickers. Trials were recorded and automatically scored by Ethovision 12 (Noldus Information Technology). Acquisition training consisted of 4 trials a day for 5 days. Each training trial began by lowering the mouse into the water close to the pool edge, in a quadrant that was either right of, left of, or opposite to, the target quadrant containing the platform. The start location for each trial was alternated in a semi-random order for each mouse. The hidden platform remained in the same quadrant for all trials during acquisition training for a given mouse but varied across subject mice. Mice were allowed a maximum of 60 s to reach the platform. A mouse that failed to reach the platform in 60 s was guided to the platform by the experimenter. Mice were left on the platform for 15 s before being removed. After each trial, the subject was placed in a cage lined with absorbent paper towels and allowed to rest under an infrared heating lamp for 60 s. Two hours after the completion of training on day 5, the platform was removed, and mice were tested in a 60 s probe trial. Parameters recorded during training days were latency to reach the platform, total distance traveled, and swim speed. Time spent in each quadrant and number of crossings over the trained platform location and over analogous locations in the other quadrants were used to analyze probe trial performance.

### UV crosslinking immunoprecipitation and sequencing (CLIP-seq) analysis

CLIP-seq experiments were conducted with hearts from two-week-old WT mice using anti-hnRNP U (A300-690A, Bethyl Laboratories). Sample preparation, crosslinked-RNA recovery, library preparation and sequencing were performed according to published protocols [81]. After linker sequence trimming and duplication collapsing, CLIP reads were aligned to mouse genome (mm10) by Novoalign, and unique tags were clustered. Distribution of CLIP tags to different genomic regions were determined. We obtained 822,984 unique tags for hnRNP U with a majority (63%) of these tags mapped to introns, 8% mapped to exons and the remaining tags mapped to promoter, 5’ and 3’ UTRs and intergenic regions.

### Single-cell RNA-sequencing and data integration

Neocortical and hippocampal tissue was dissected from postnatal day 0 pups and subjected to a papain dissociation. Following papain dissociation and tissue trituration, all neocortical and hippocampal samples were filtered through a 40 μm cell strainer to enrich for single cells in the resulting suspension. Cell viability was subsequently assessed, with a cutoff of 70% or greater to be used for sequencing. Single cell RNA-seq libraries were constructed using the 10X Chromium Single Cell 3′ Reagent Kits v2 according to manufacturer descriptions, and samples were sequenced on a NovaSeq 6000. Reads were aligned to the mm10 genome using the 10X CellRanger pipeline with default parameters to generate the feature-barcode matrix.

We used Seurat v3 to perform downstream QC and analyses on feature-barcode matrices [53,54]. We removed all genes that were not detected in at least 4 cells. We further removed cells with fewer than 1,000 genes or more than 5,000 genes detected. For cortical cells, we removed all cells with greater than 8% of reads mapping to mitochondrial genes. For hippocampal cells, we removed all cells with greater than 15% of reads mapping to mitochondrial genes. The filtered matrices were log-normalized and scaled to 10,000 transcripts per cell. We used the variance-stabilizing transformation implemented in the FindVariableFeatures function to identify the top 2,000 most variable genes per sample. We used Seurat’s data integration method to harmonize gene expression across datasets prior to clustering. We first identified anchors between samples in each dataset using the FindIntegrationAnchors function, which uses canonical correlation analysis (CCA) to identify pairwise cell correspondences between samples. We then computed an integrated expression matrix using these anchors as input to the IntegrateData function.

Next, we used linear regression to regress out the number of UMIs per cell and percentage of mitochondrial reads using the ScaleData function on the integrated expression matrices. We then performed dimensionality reduction using PCA. For each dataset, we selected the top 30 dimensions to compute a cellular distance matrix, which was used to generate a K-nearest neighbor graph. The KNN was used as input to the Louvain Clustering algorithm implemented in the FindClusters function. For clustering via Louvain, we chose a resolution parameter of 0.8. We visualized the cells using UMAP via the RunUMAP function.

To annotate and merge clusters, we performed differential gene expression analysis on the integrated expression values between each cluster using the default parameters in the FindMarkers function, which implements a Wilcoxon test and corrects p-values via Bonferroni correction. Additionally, we visualized the expression of canonical marker genes aggregated from previous single-cell publications [82–85]. Clusters representing microglia and smooth muscle cells were excluded given limited (5–10) cells represented from these cell types.

### Differential gene expression analysis

We performed cell-type-specific differential gene expression analysis using MAST [86], as implemented in Seurat’s FindMarkers function, to identify genes dysregulated between mutant and wildtype cells. We excluded all non-coding genes, genes encoding ribosomal proteins, and pseudogenes from our analysis to reduce the multiple testing burden. For each cell type, we fit a linear mixed model that included the gene detection rate (ngeneson) and gender as latent variables:

$$\text{zlm}\left(\sim \text{ngeneson}+\text{gender}\right)$$

We corrected the p-values using the Benjamini-Hochberg FDR method. We considered genes with a log2(fold change) value of at least 0.14 (10% difference) and FDR < 0.05 as differentially expressed. We performed gene ontology analysis using g:Profiler [87], using all tested genes per cell type as a background set. P-values were generated using Fisher’s Exact Test and corrected via FDR.

For the burden analysis, we down sampled the data to include 300 cells per population. To increase power, we pooled cells from male and female samples. We ran the differential expression analysis as described above, removing gender as a latent variable.

For disease gene enrichment analyses, human homologs for all tested mouse genes were obtained using biomaRt. Significant and nonsignificant mouse genes were annotated based on the respective human homolog disease gene status. Genes without human homologs were not used in the analysis. The epilepsy-associated gene list was based on a prior publication [88]. Autism genes were based on SFARI genes with gene scores 1, 2, 3 and S on SFARI.org [89]. Confirmed monoallelic developmental delay genes (obtained in 2019) were obtained from the Deciphering Developmental Disorders study [90]. A Fisher’s exact test was performed on significant downregulated and upregulated DEGs compared to all non-significant DEGs.

### Transcriptomic reversal

We queried the Connectivity Map (clue.io) to identify compounds most likely to reverse disease expression signatures [57,58]. Our disease expression signatures included the top 150 downregulated genes per query. We compared the compounds predicted to reverse the expression profile derived from excitatory cells in the subiculum as well as the expression profile that would have been recovered via bulk RNA-sequencing. To generate this second signature (i.e. the pseudo-bulk signature), we performed differential gene expression between mutant and wild type cells using MAST without factoring in cell types. We considered compounds that achieved a Connectivity Score of less than -90 as most likely to reverse the disease signatures.

## Supporting information

S1 Fig HnRNP U is expressed ubiquitously in neocortical cells. Primary neocortical cell cultures at day *in vitro* 9 showing hnRNP U (red) co-staining with nuclear marker Dapi (blue) and cell markers (green) **(A)** Map2 (neuronal), **(B)** Gad67 (inhibitory), **(C)** Ctip2 (deeper layer pyramidal neurons), **(D)** Satb2 (upper layer pyramidal neurons) and **(E)** Gfap (astrocytes). Scale bar = 50μm.
(TIF)

S2 Fig HnRNP U is expressed ubiquitously in hippocampal cells. hnRNP U (red) co-staining with nuclear marker Dapi and cell markers (green) **(A)** Map2 (neuronal), **(B)** Gad67 (inhibitory), **(C)** Ctip2 (deeper layer pyramidal neurons), **(D)** Satb2 (upper layer pyramidal neurons) and **(E)** Gfap (astrocytes). Scale bar = 50μm.
(TIF)

S3 Fig Generation of a heterozygous *Hnrnpu* knockout mouse model. **(A)** A representative genotyping agarose gel showing the 113-bp deletion. **(B)** Western blot image using antibody targeting hnRNP U C-terminus **(C)** Western blot image using an antibody targeting hnRNP U N-terminus.
(TIF)

S4 Fig No overt morphological defects observed in *Hnrnpu*^+/113DEL^ mice. **(A)** Representative H&E-stained coronal sections of WT and HET brains at PND0. Black arrows indicate the corpus callosum. Level of sections indicated by the respective cartoons. Scale bar = 1 mm. **(B)** Brain width, height, cortical thickness and hippocampal width were measured from caudal sections and normalized to respective body weight (n = 3 animals per genotype). Bonferroni-corrected T-test p> 0.99 for each test (width t = 0.79, height t = 0.25, cortical thickness t = 1.2, hippocampus width t = 0.39, df = 4). Error bars = SEM.
(TIF)

S5 Fig Delayed physical and sensorimotor development in *Hnrnpu*^+/113DEL^ mice. **(A)** Body weight of PND0 pups, also stratified by sex. Unpaired t-test for all mice p = 3.9x10^-3^ (two-tailed, t = 3.15, df = 27), for males p = 0.01 (t = 2.82, df = 14) and for females p = 0.02 (t = 2.53, df = 15). **(B)** Pup weights stratified by sex (males = 10 WT, 13 HET; females = 6 WT, 3 HET). Permuted MWU p-values for males: PND4 & 6 = 1x10^-4^, PND8 = 7x10^-4^, PND10 = 6.4x10^-3^, PND12 = 0.03, and for females: PND4-12 = <1x10^-4^. **(C)** Adult body weights (n = 9 per genotype). Permuted Mann-Whitney U (MWU) p-values for all mice: wk4 = 2x10^-4^, wk5 & 6< 1x10^-4^, wk7 = 4x10^-4^, wk8 = 3x10^-4^, wk9 = 1.1x10^-3^, wk10< 1x10^-4^. **(D)** Adult weight stratified by sex. Permuted MWU p-values for males: wk4 = 0.20, wk5 = 0.11, wk6 = 0.06, wk7 = 0.06, wk8 = 0.06, wk9 = 0.06, wk10 = 0.05, and for females: wk4 = 9.0x10^-3^, wk5-10 <1x10^-4^. **(E)** Representative image of 8 wk adult male WT and HET. **(F)** Vertical screen test. Permuted MWU p-values: PND4 = 0.31, PND6 = 0.05, PND8 = 0.07, PND10 = 0.07, PND12 = 0.96. **(G)** Surface righting test. Permuted MWU p-values: PND4 = 0.3, PND6 = 0.42, PND8 = 0.07, PND10 = 7x10^-4^, PND12 = 0.15. **(H)** 90° negative geotaxis test. Permuted MWU p-values: PND4 = 0.96, PND6 = 0.91, PND8 = 0.14, PND10 = 0.16, PND12 = 4.2x10^-3^. **(I)** 180° negative geotaxis test. Permuted MWU p-values: PND4 = 0.39, PND6 = 0.2, PND8 = 0.16, PND10 = 0.47, PND12 = 0.08. (C-F, n = 16 per genotype). **(J)** USV’s stratified by sex (males = 12 WT, 13 HET; females = 5 WT, 3 HET). Permuted MWU p-values males: PND3 = 0.36, PND5&7< 1x10^-4^, PND9 = 0.01, PND11 = 1x10^-3^; females: PND3 = 0.40, PND5 = 0.57, PND7< 1x10^-4^, PND9 = 0.58, PND11 = 0.16. PND = postnatal day. Error bars = SEM.
(TIF)

S6 Fig Adult behavior studies of *Hnrnpu*^+/113DEL^ mice. **(A)** Summary table of EEG data. Sz = seizure **(B)** Elevated plus maze (EPM) percent time spent in open arm (n = 14 WT, 22 HET). Welch’s t-test p = 0.47 (two-tailed, t = 0.73, df = 33.8). **(C)** EPM total arm entries (n = 14 WT, 22 HET). T-test p = 0.12 (two-tailed, t = 1.58, df = 34). **(D)** Open field ambulatory distance divided into six 10 min bins (n = 16 WT, 18 HET). Two-way repeated-measure (RM) ANOVA p = 0.026 (f = 5.4, df = 1). **(E)** Time spent in the center of open field, divided into 10 min bins (n = 16 WT, 18 HET). Two-way RM ANOVA p = 0.09 (f = 3.06, df = 1). **(F)** Catwalk front-paw (FP) and hind-paw (HP) print area (n = 17 WT, 19 HET). Unpaired, two-tailed t-test FP p = 0.024 (t = 2.4, df = 34), HP p< 1x10^-4^ (t = 4.4, df = 34). **(G)** Catwalk max contact max intensity (MCMI) of FP and HP (n = 17 WT, 19 HET). Mann-Whitney U (MWU) FP p = 0.08, HP p< 1x10^-4^. **(H)** Representative images of FP and HP prints of WT and HET mice. **(I)** Catwalk base of support for FP and HP (n = 17 WT, 19 HET). MWU FP p = 0.08, HP p = 1.5x10^-3^. **(J)** Morris Water Maze (MWM) latency to platform (acquisition period) measured over 5 consecutive days (n = 15 WT, 18 HET). Two-way RM ANOVA p = 0.22 (f = 1.56, df = 1). **(K)** MWM swim distance measured over 5 days (n = 15 WT, 18 HET). Two-way RM ANOVA p = 0.1 (f = 2.88, df = 1). **(L)** MWM probe trial measured as time spent in each quadrant of the pool (n = 15 WT, 18 HET). One-way RM ANOVA on ranks: WT T (trained quadrant) vs L (left quadrant) p< 1x10^-3^, T vs R (right quadrant) p = 0.2, T vs opp (opposite quadrant) p< 1x10^-3^. HET T vs R p = 2x10^-3^, T vs L p = 0.64, T vs opp p = 0.42. **(M)** Y-Maze percent duration spent in novel vs familiar arm (n = 16 WT, 18 HET). One-way RM ANOVA WT p = 5x10^-3^ (f = 10.5, df = 1), HET p = 3x10^-3^ (f = 11.5, df = 1). **(N)** Y-Maze percent spontaneous alternation (n = 16 WT, 18 HET). Welch’s t-test p = 0.86 (two-tailed, t = 0.18, df = 20.9). **(O)** Fear conditioning percent freezing (n = 16 WT, 18 HET). MWU post-train p = 7x10^-4^, context p< 1x10^-4^, pre-cue p = 0.02, cued p = 1.8x10^-3^. **(P)** Startle response (y-axis) across different decibels (x-axis) (n = 11 WT, 13 HET). T-test p = 0.96. Error bars = SEM.
(TIF)

S7 Fig Abnormal low gamma oscillations in *Hnrnpu* mutant mice. **(A**) A representative example of EEG signals in a *Hnrnpu* heterozygous mutant mouse (Het). From top to bottom: EEG spectrogram (20-50Hz), raw EEG traces (10 min in total), brain states (gray for wake, orange for slow-wave sleep, or SWS), and EEG traces in wake and SWS (3 s, enlarged from a1 and a2). (**B**) A representative example of EEG signals in a wildtype (WT) mouse. EEG traces in the bottom are enlarged from b1 and b2 positions. (**C**) Spectral analysis of EEG during wake and SWD in a recording session in *Hnrnpu* mutant and wildtype mice. (**D**) Quantitation of EEG low gamma power in *Hnrnpu* mutant and wildtype mice (6 recording sessions in 4 mutant and 4 wildtype mice, each session is 24 hours. * P<0.05, ns, no significance, unpaired t-test).
(TIF)

S8 Fig Single-cell RNA-seq dataset integration. **(A)** UMAP plots of cells from each hippocampal sample. **(B)** UMAP plots of cells from each cortical sample. Het_F: neocortical cells from a female; Het_M: neocortical cells from a male. Het_F = *Hnrnpu*^+/113DEL^ female; Het_M = *Hnrnpu*^+/113DEL^ male; WT_F = wildtype female; WT_M = wildtype male.
(TIF)

S9 Fig Number of cells per cluster. **(A, B)** Number of cells detected in each cell population, colored by sample. **(A)** represents hippocampal clusters and **(B)** represents neocortical clusters. Het_F = *Hnrnpu*^+/113DEL^ female; Het_M = *Hnrnpu*^+/113DEL^ male; WT_F = wildtype female; WT_M = wildtype male.
(TIF)

S10 Fig Transcriptomic signature reversal for hippocampal disease signatures. **(A, B)** Distribution of Connectivity Scores for the subiculum-derived and pseudo-bulk derived disease expression signatures. **(C, D)** Top 20 compounds predicted to reverse the subiculum and pseudo-bulk signatures. Compounds in red represent compounds prioritized for both signatures. **(E)** Venn Diagram depicting the overlap between all compounds achieving a Connectivity Score less than -90 for the subiculum and pseudo-bulk signatures.
(TIF)

S1 Table List of human *HNRNPU* pathogenic variants. (PDF)

S2 Table Canonical cell-type-specific markers used to annotate cell clusters. (XLSX)

S3 Table Hippocampal differential gene expression results for all cell types. (XLSX)

S4 Table Neocortical differential gene expression results for all cell types. (XLS)

S5 Table Gene ontology results for hippocampal and neocortical up and downregulated genes. (XLSX)

S6 Table Compounds with Connectivity Scores less than -90 for subiculum-derived and pseudobulk transcriptional signatures. (XLSX)

S1 Data Numerical values underlying graphs and summary statistics. (XLSX)
