# Supplementary figures and images for: Neurodevelopmental deficits and cell-type-specific transcriptomic perturbations in a mouse model of HNRNPU haploinsufficiency
Source: PLoS Genet. 2023 Oct 2;19(10):e1010952. doi: 10.1371/journal.pgen.1010952 (PMC10569524; doi:10.1371/journal.pgen.1010952)

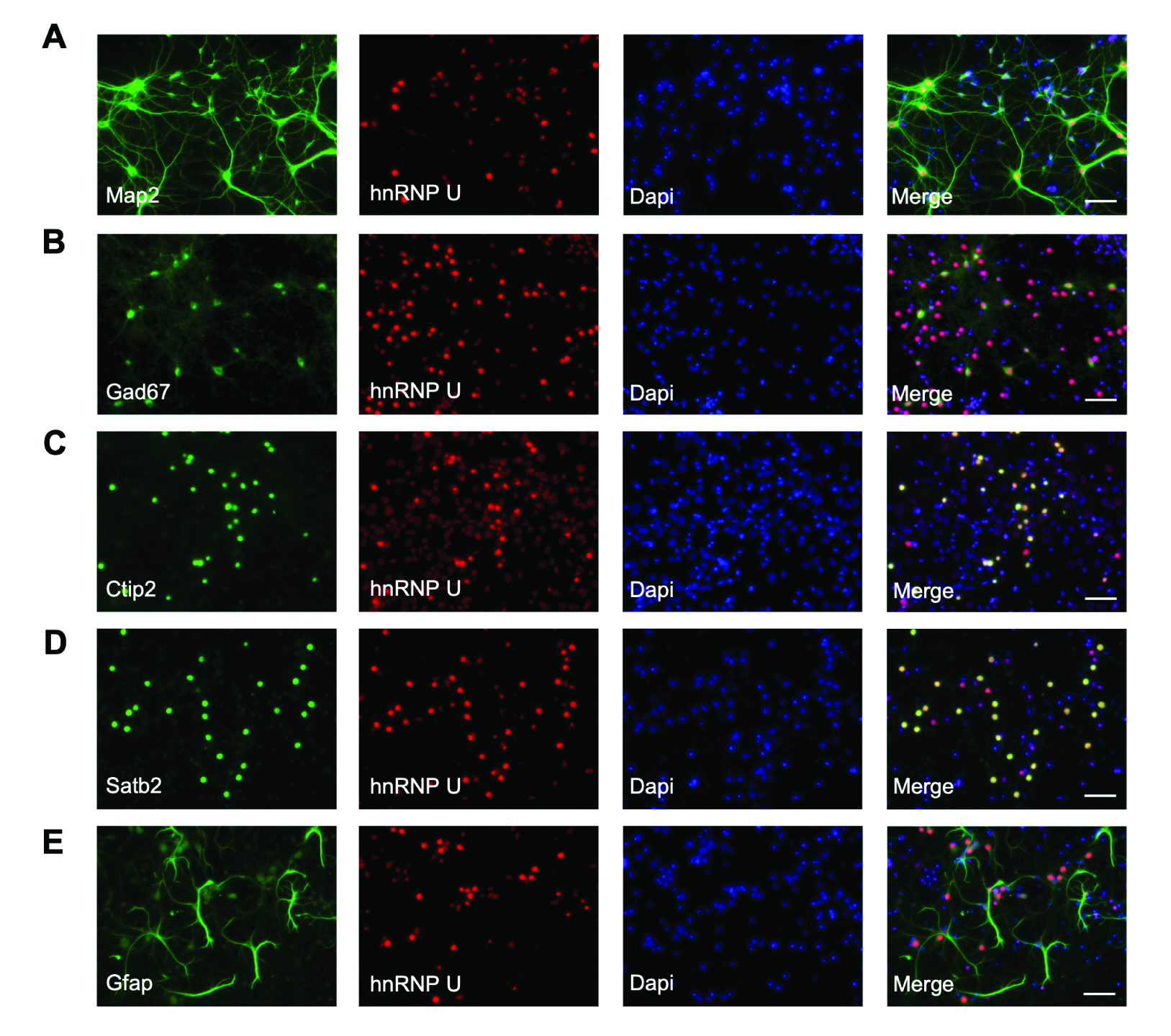

Supplement: S1 Fig — Primary neocortical cell cultures at day in vitro 9 showing hnRNP U (red) co-staining with nuclear marker Dapi (blue) and cell markers (green) (A) Map2 (neuronal), (B) Gad67 (inhibitory), (C) Ctip2 (deeper layer pyramidal neurons), (D) Satb2 (upper layer pyramidal neurons) and (E) Gfap (astrocytes). Scale bar = 50μm. (TIF) [file pgen.1010952.s001.tif]

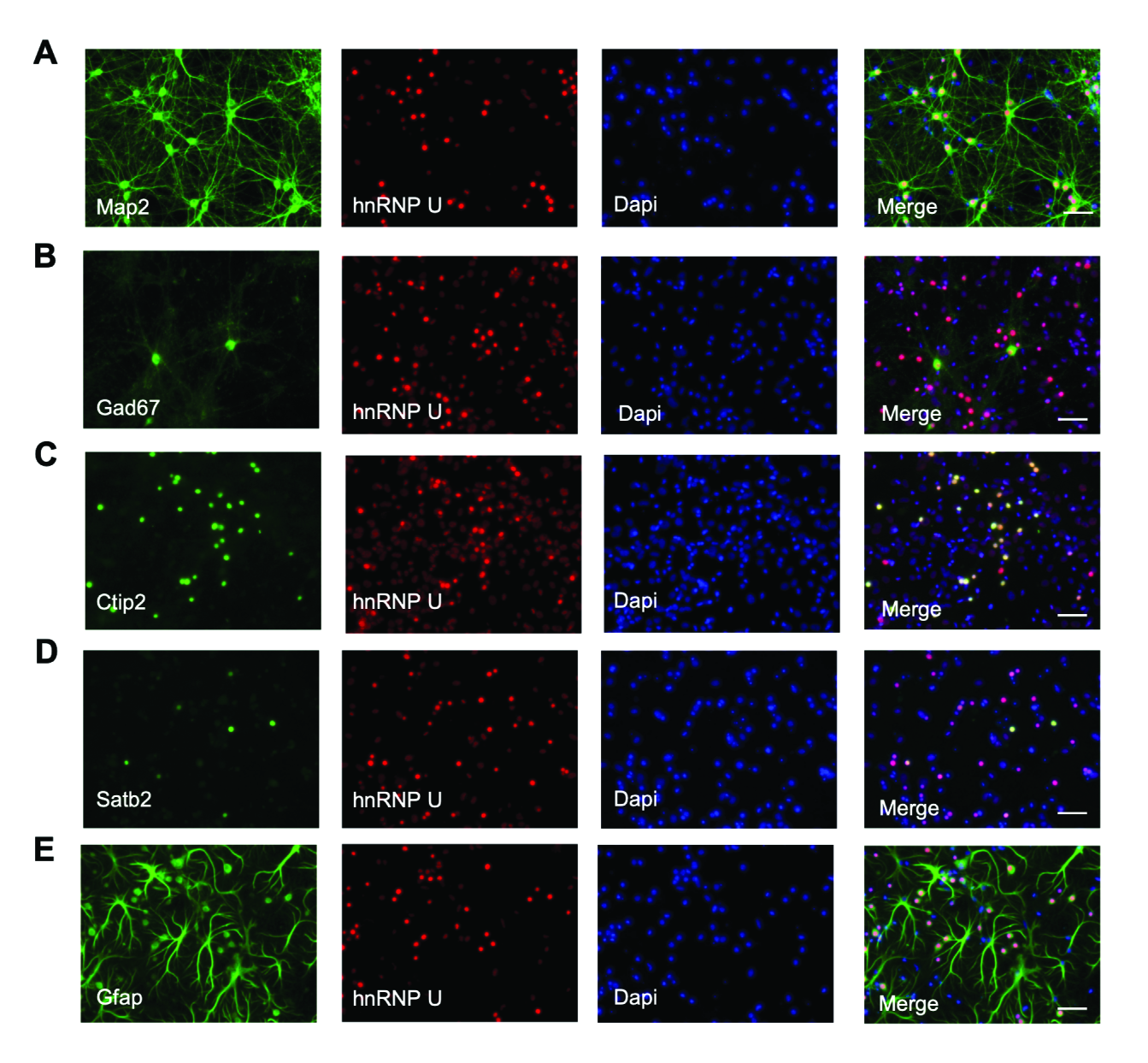

Supplement: S2 Fig — hnRNP U (red) co-staining with nuclear marker Dapi and cell markers (green) (A) Map2 (neuronal), (B) Gad67 (inhibitory), (C) Ctip2 (deeper layer pyramidal neurons), (D) Satb2 (upper layer pyramidal neurons) and (E) Gfap (astrocytes). Scale bar = 50μm. (TIF) [file pgen.1010952.s002.tif]

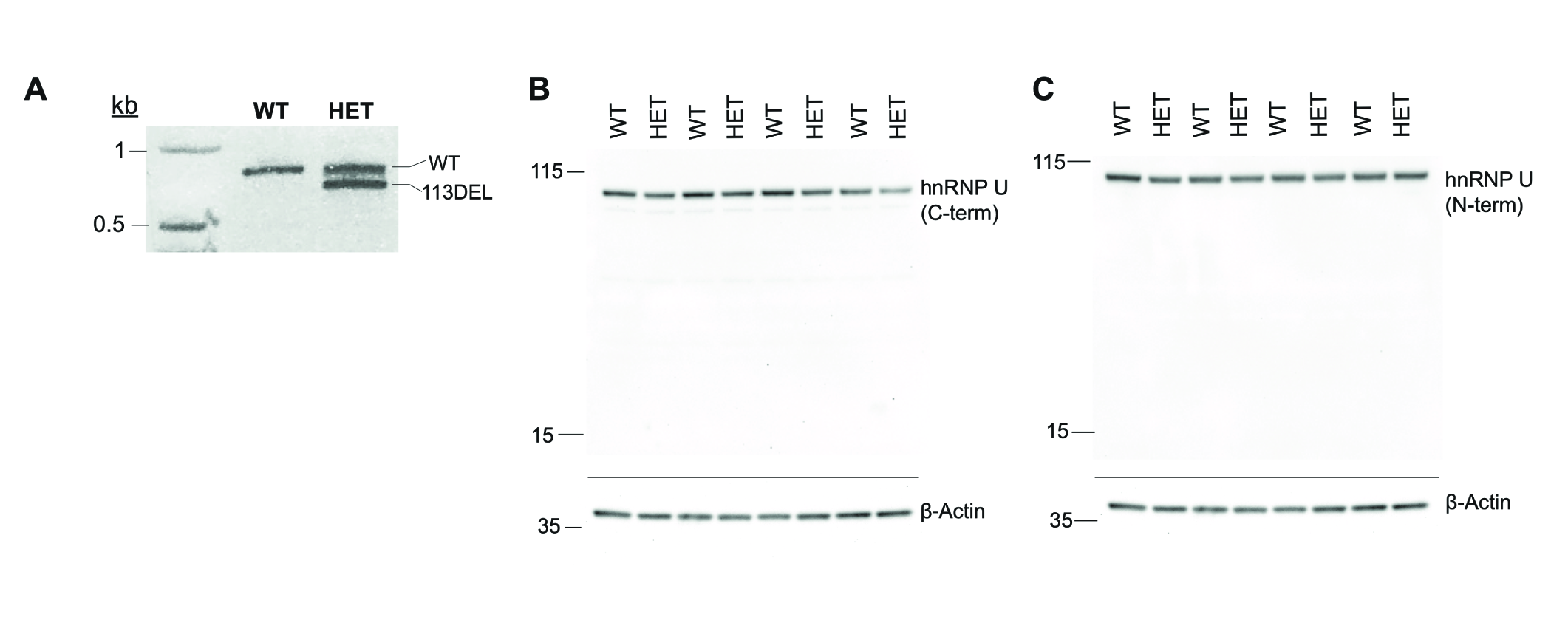

Supplement: S3 Fig — (A) A representative genotyping agarose gel showing the 113-bp deletion. (B) Western blot image using antibody targeting hnRNP U C-terminus (C) Western blot image using an antibody targeting hnRNP U N-terminus. (TIF) [file pgen.1010952.s003.tif]

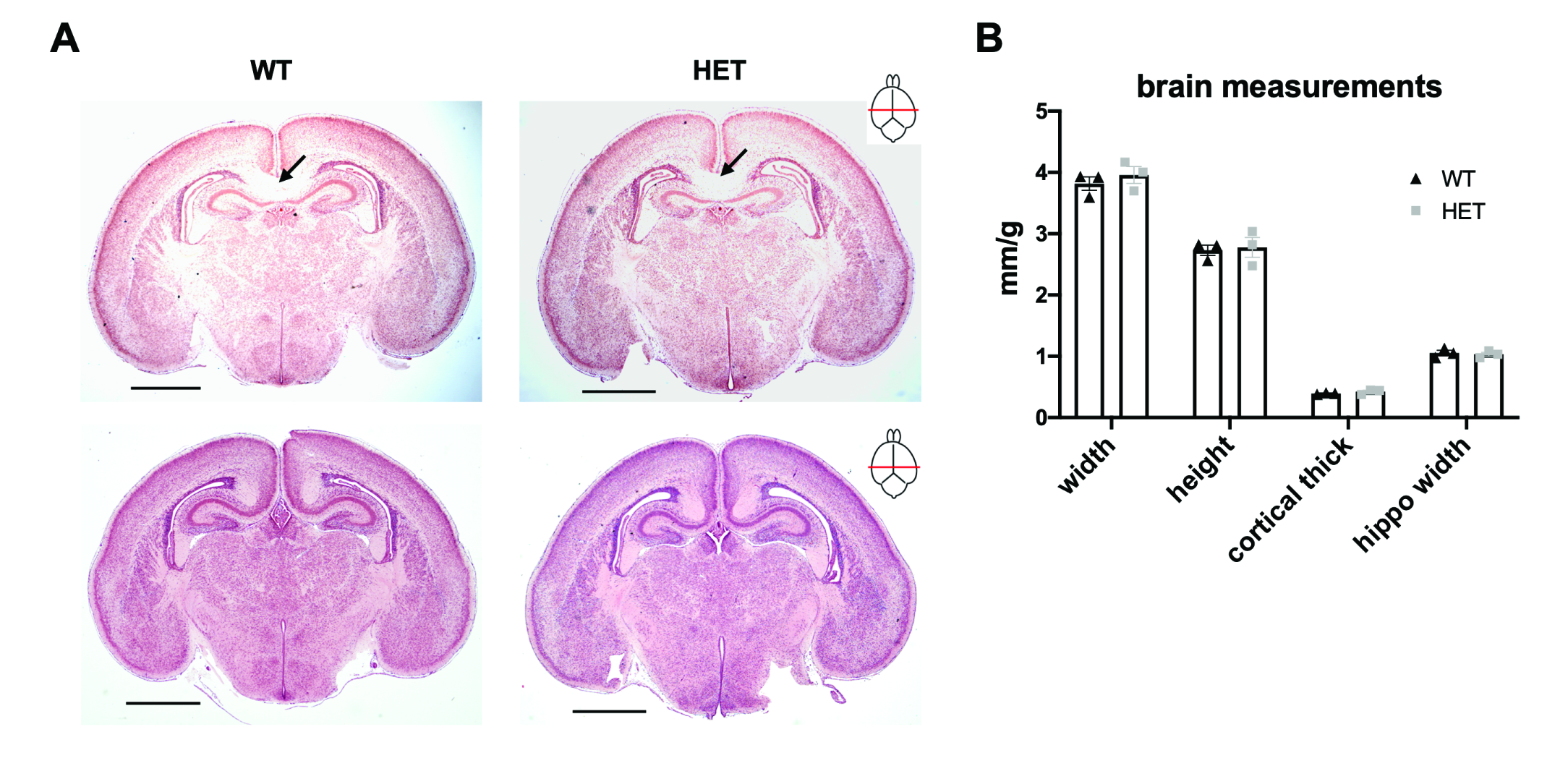

Supplement: S4 Fig — (A) Representative H&E-stained coronal sections of WT and HET brains at PND0. Black arrows indicate the corpus callosum. Level of sections indicated by the respective cartoons. Scale bar = 1 mm. (B) Brain width, height, cortical thickness and hippocampal width were measured from caudal sections and normalized to respective body weight (n = 3 animals per genotype). Bonferroni-corrected T-test p> 0.99 for each test (width t = 0.79, height t = 0.25, cortical thickness t = 1.2, hippocampus width t = 0.39, df = 4). Error bars = SEM. (TIF) [file pgen.1010952.s004.tif]

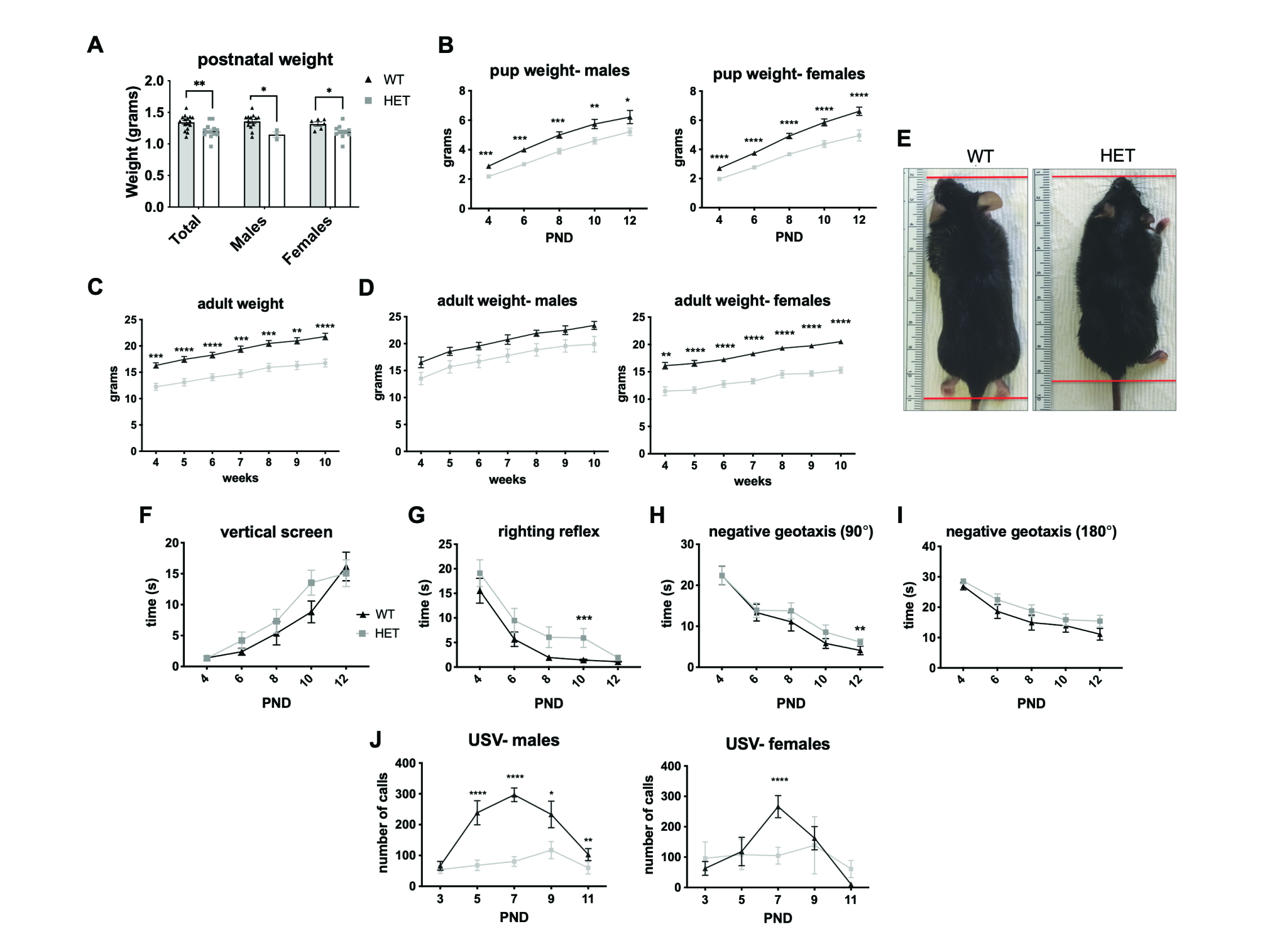

Supplement: S5 Fig — (A) Body weight of PND0 pups, also stratified by sex. Unpaired t-test for all mice p = 3.9x10^-3^ (two-tailed, t = 3.15, df = 27), for males p = 0.01 (t = 2.82, df = 14) and for females p = 0.02 (t = 2.53, df = 15). (B) Pup weights stratified by sex (males = 10 WT, 13 HET; females = 6 WT, 3 HET). Permuted MWU p-values for males: PND4 & 6 = 1x10^-4^, PND8 = 7x10^-4^, PND10 = 6.4x10^-3^, PND12 = 0.03, and for females: PND4-12 = <1x10^-4^. (C) Adult body weights (n = 9 per genotype). Permuted Mann-Whitney U (MWU) p-values for all mice: wk4 = 2x10^-4^, wk5 & 6< 1x10^-4^, wk7 = 4x10^-4^, wk8 = 3x10^-4^, wk9 = 1.1x10^-3^, wk10< 1x10^-4^. (D) Adult weight stratified by sex. Permuted MWU p-values for males: wk4 = 0.20, wk5 = 0.11, wk6 = 0.06, wk7 = 0.06, wk8 = 0.06, wk9 = 0.06, wk10 = 0.05, and for females: wk4 = 9.0x10^-3^, wk5-10 <1x10^-4^. (E) Representative image of 8 wk adult male WT and HET. (F) Vertical screen test. Permuted MWU p-values: PND4 = 0.31, PND6 = 0.05, PND8 = 0.07, PND10 = 0.07, PND12 = 0.96. (G) Surface righting test. Permuted MWU p-values: PND4 = 0.3, PND6 = 0.42, PND8 = 0.07, PND10 = 7x10^-4^, PND12 = 0.15. (H) 90° negative geotaxis test. Permuted MWU p-values: PND4 = 0.96, PND6 = 0.91, PND8 = 0.14, PND10 = 0.16, PND12 = 4.2x10^-3^. (I) 180° negative geotaxis test. Permuted MWU p-values: PND4 = 0.39, PND6 = 0.2, PND8 = 0.16, PND10 = 0.47, PND12 = 0.08. (C-F, n = 16 per genotype). (J) USV’s stratified by sex (males = 12 WT, 13 HET; females = 5 WT, 3 HET). Permuted MWU p-values males: PND3 = 0.36, PND5&7< 1x10^-4^, PND9 = 0.01, PND11 = 1x10^-3^; females: PND3 = 0.40, PND5 = 0.57, PND7< 1x10^-4^, PND9 = 0.58, PND11 = 0.16. PND = postnatal day. Error bars = SEM. (TIF) [file pgen.1010952.s005.tif]

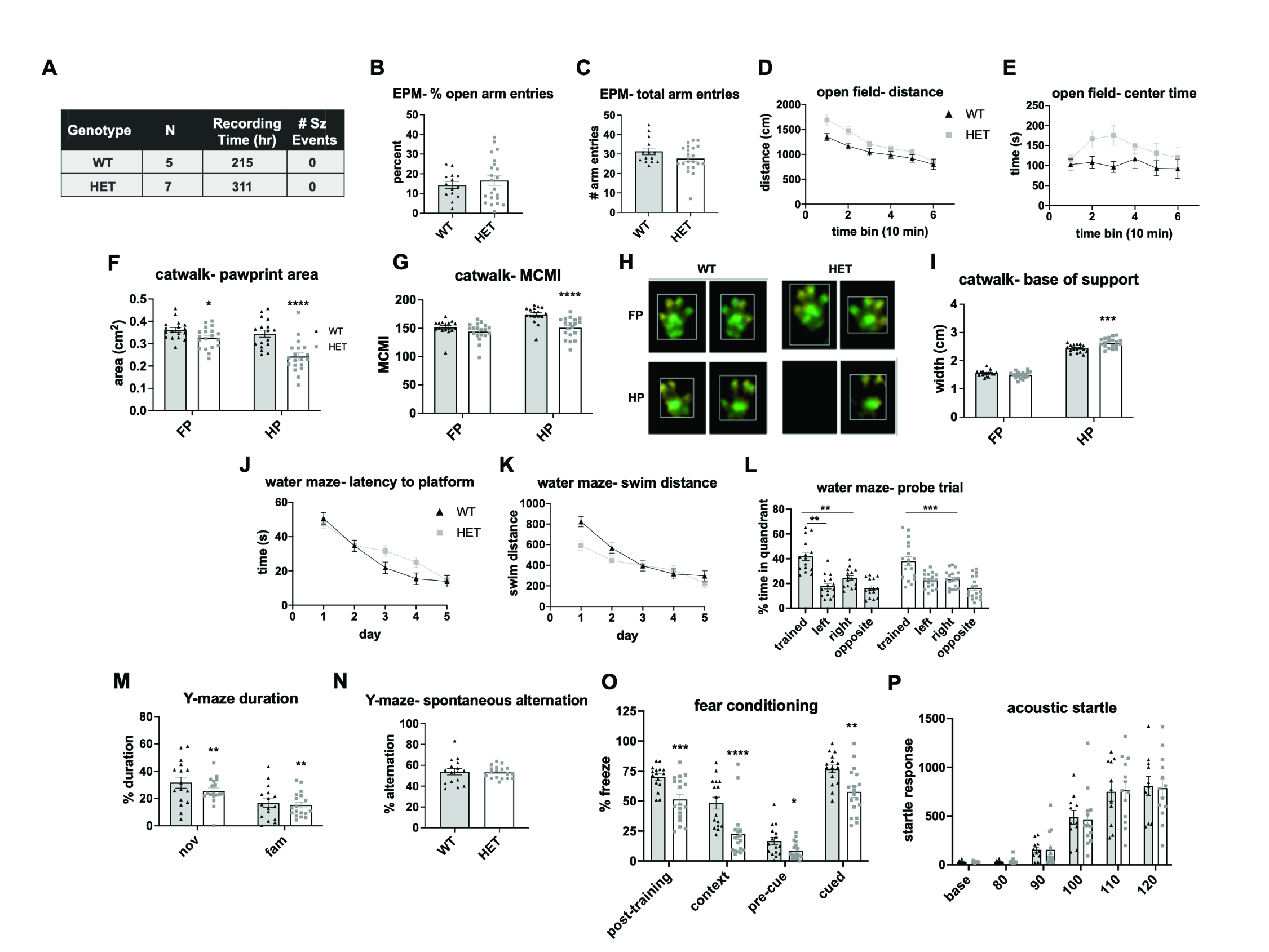

Supplement: S6 Fig — (A) Summary table of EEG data. Sz = seizure (B) Elevated plus maze (EPM) percent time spent in open arm (n = 14 WT, 22 HET). Welch’s t-test p = 0.47 (two-tailed, t = 0.73, df = 33.8). (C) EPM total arm entries (n = 14 WT, 22 HET). T-test p = 0.12 (two-tailed, t = 1.58, df = 34). (D) Open field ambulatory distance divided into six 10 min bins (n = 16 WT, 18 HET). Two-way repeated-measure (RM) ANOVA p = 0.026 (f = 5.4, df = 1). (E) Time spent in the center of open field, divided into 10 min bins (n = 16 WT, 18 HET). Two-way RM ANOVA p = 0.09 (f = 3.06, df = 1). (F) Catwalk front-paw (FP) and hind-paw (HP) print area (n = 17 WT, 19 HET). Unpaired, two-tailed t-test FP p = 0.024 (t = 2.4, df = 34), HP p< 1x10^-4^ (t = 4.4, df = 34). (G) Catwalk max contact max intensity (MCMI) of FP and HP (n = 17 WT, 19 HET). Mann-Whitney U (MWU) FP p = 0.08, HP p< 1x10^-4^. (H) Representative images of FP and HP prints of WT and HET mice. (I) Catwalk base of support for FP and HP (n = 17 WT, 19 HET). MWU FP p = 0.08, HP p = 1.5x10^-3^. (J) Morris Water Maze (MWM) latency to platform (acquisition period) measured over 5 consecutive days (n = 15 WT, 18 HET). Two-way RM ANOVA p = 0.22 (f = 1.56, df = 1). (K) MWM swim distance measured over 5 days (n = 15 WT, 18 HET). Two-way RM ANOVA p = 0.1 (f = 2.88, df = 1). (L) MWM probe trial measured as time spent in each quadrant of the pool (n = 15 WT, 18 HET). One-way RM ANOVA on ranks: WT T (trained quadrant) vs L (left quadrant) p< 1x10^-3^, T vs R (right quadrant) p = 0.2, T vs opp (opposite quadrant) p< 1x10^-3^. HET T vs R p = 2x10^-3^, T vs L p = 0.64, T vs opp p = 0.42. (M) Y-Maze percent duration spent in novel vs familiar arm (n = 16 WT, 18 HET). One-way RM ANOVA WT p = 5x10^-3^ (f = 10.5, df = 1), HET p = 3x10^-3^ (f = 11.5, df = 1). (N) Y-Maze percent spontaneous alternation (n = 16 WT, 18 HET). Welch’s t-test p = 0.86 (two-tailed, t = 0.18, df = 20.9). (O) Fear conditioning percent freezing (n = 16 WT, 18 HET). MWU post-train p = 7x10^-4^, context p< [file pgen.1010952.s006.tif]

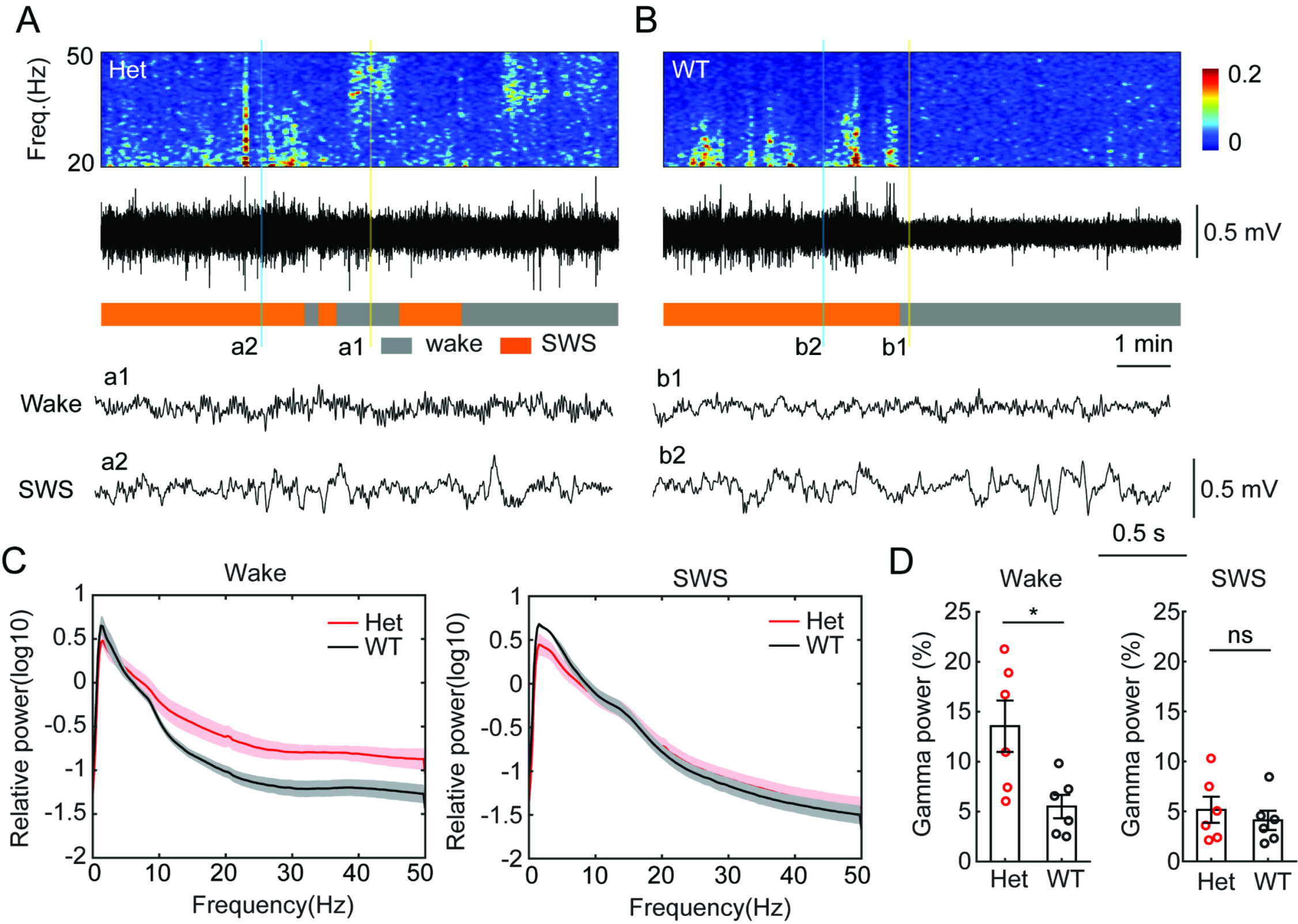

Supplement: S7 Fig — (A) A representative example of EEG signals in a Hnrnpu heterozygous mutant mouse (Het). From top to bottom: EEG spectrogram (20-50Hz), raw EEG traces (10 min in total), brain states (gray for wake, orange for slow-wave sleep, or SWS), and EEG traces in wake and SWS (3 s, enlarged from a1 and a2). (B) A representative example of EEG signals in a wildtype (WT) mouse. EEG traces in the bottom are enlarged from b1 and b2 positions. (C) Spectral analysis of EEG during wake and SWD in a recording session in Hnrnpu mutant and wildtype mice. (D) Quantitation of EEG low gamma power in Hnrnpu mutant and wildtype mice (6 recording sessions in 4 mutant and 4 wildtype mice, each session is 24 hours. * P<0.05, ns, no significance, unpaired t-test). (TIF) [file pgen.1010952.s007.tif]

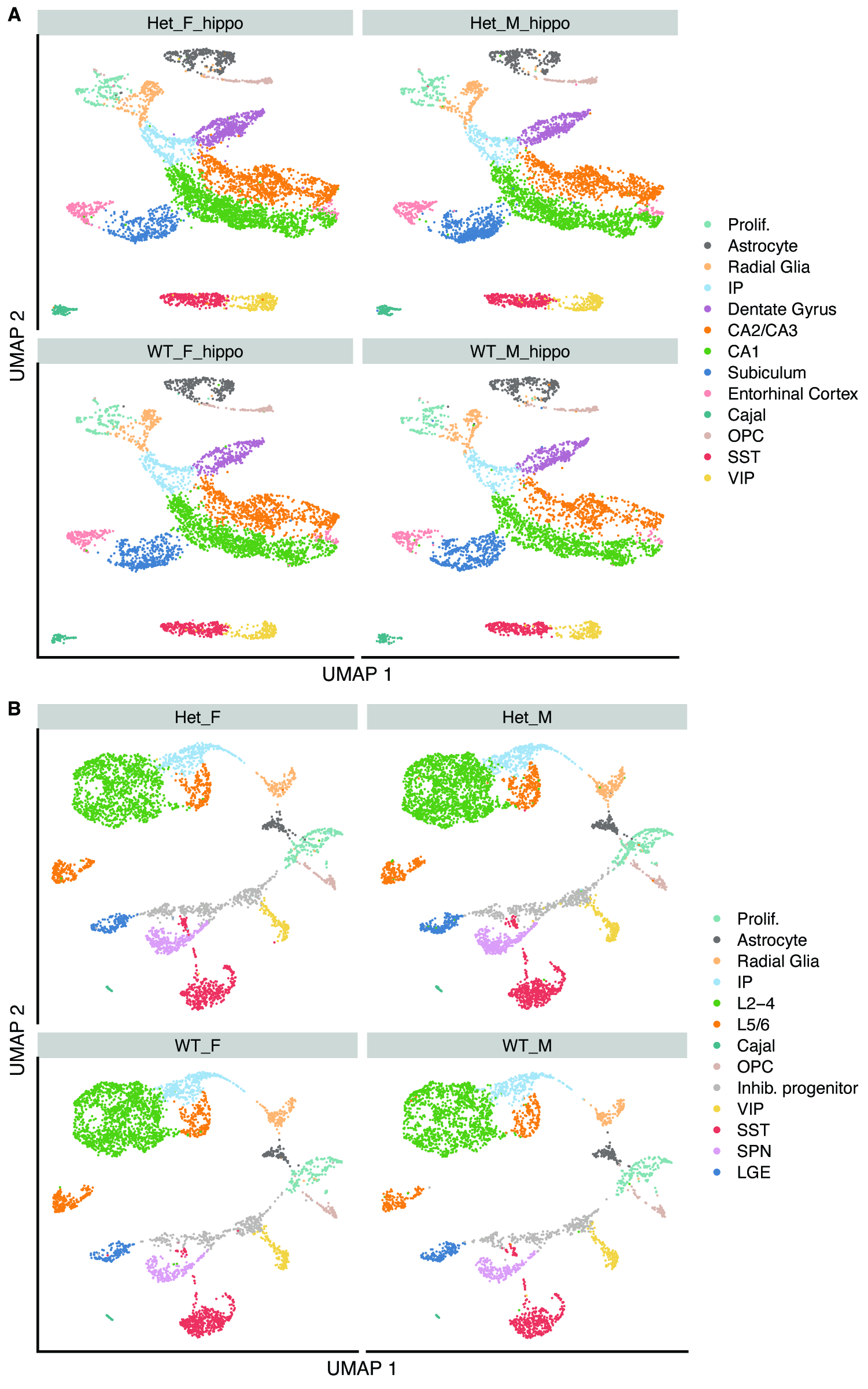

Supplement: S8 Fig — (A) UMAP plots of cells from each hippocampal sample. (B) UMAP plots of cells from each cortical sample. Het_F: neocortical cells from a female; Het_M: neocortical cells from a male. Het_F = Hnrnpu+/113DEL female; Het_M = Hnrnpu+/113DEL male; WT_F = wildtype female; WT_M = wildtype male. (TIF) [file pgen.1010952.s008.tif]

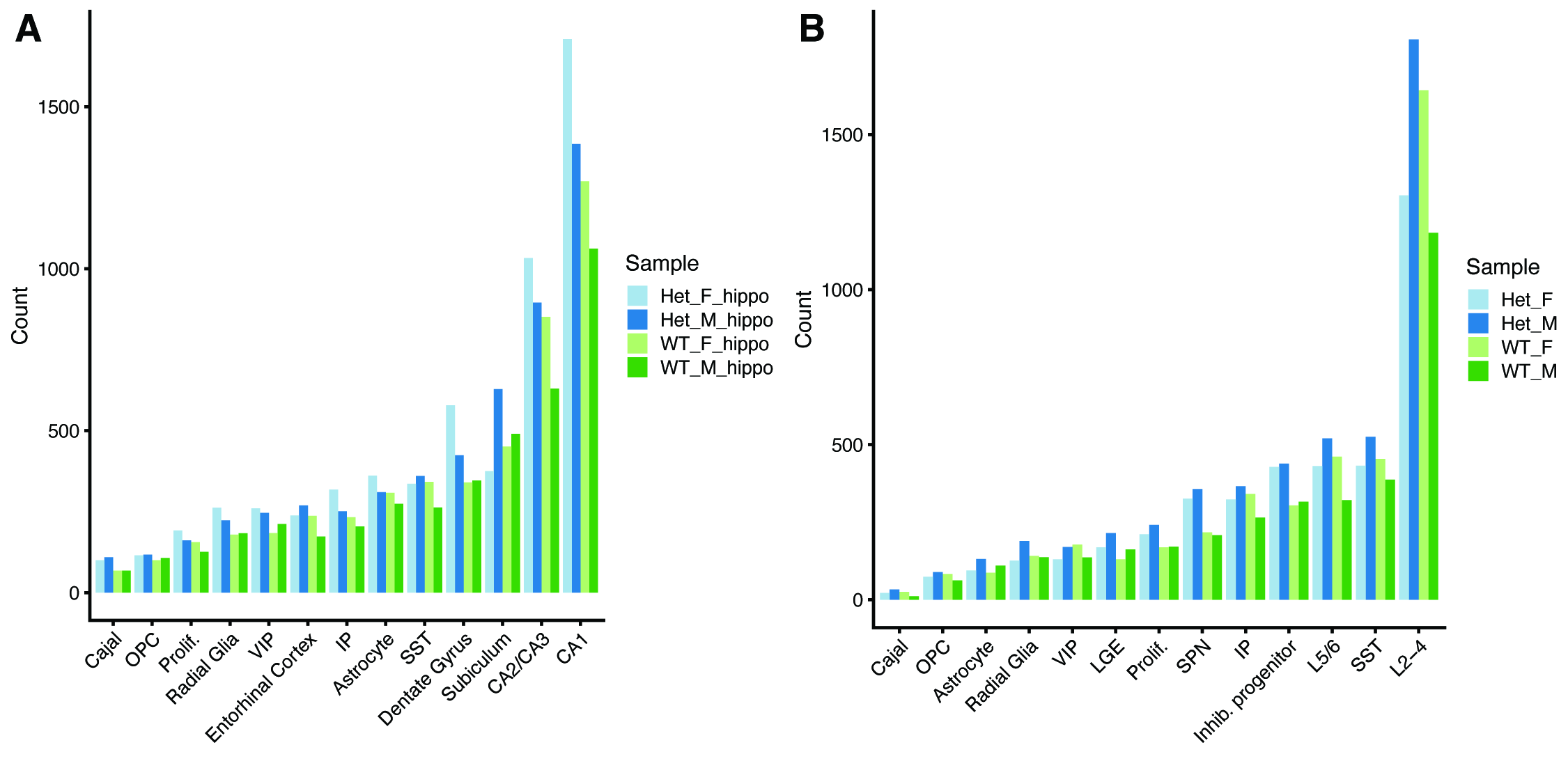

Supplement: S9 Fig — (A, B) Number of cells detected in each cell population, colored by sample. (A) represents hippocampal clusters and (B) represents neocortical clusters. Het_F = Hnrnpu+/113DEL female; Het_M = Hnrnpu+/113DEL male; WT_F = wildtype female; WT_M = wildtype male. (TIF) [file pgen.1010952.s009.tif]

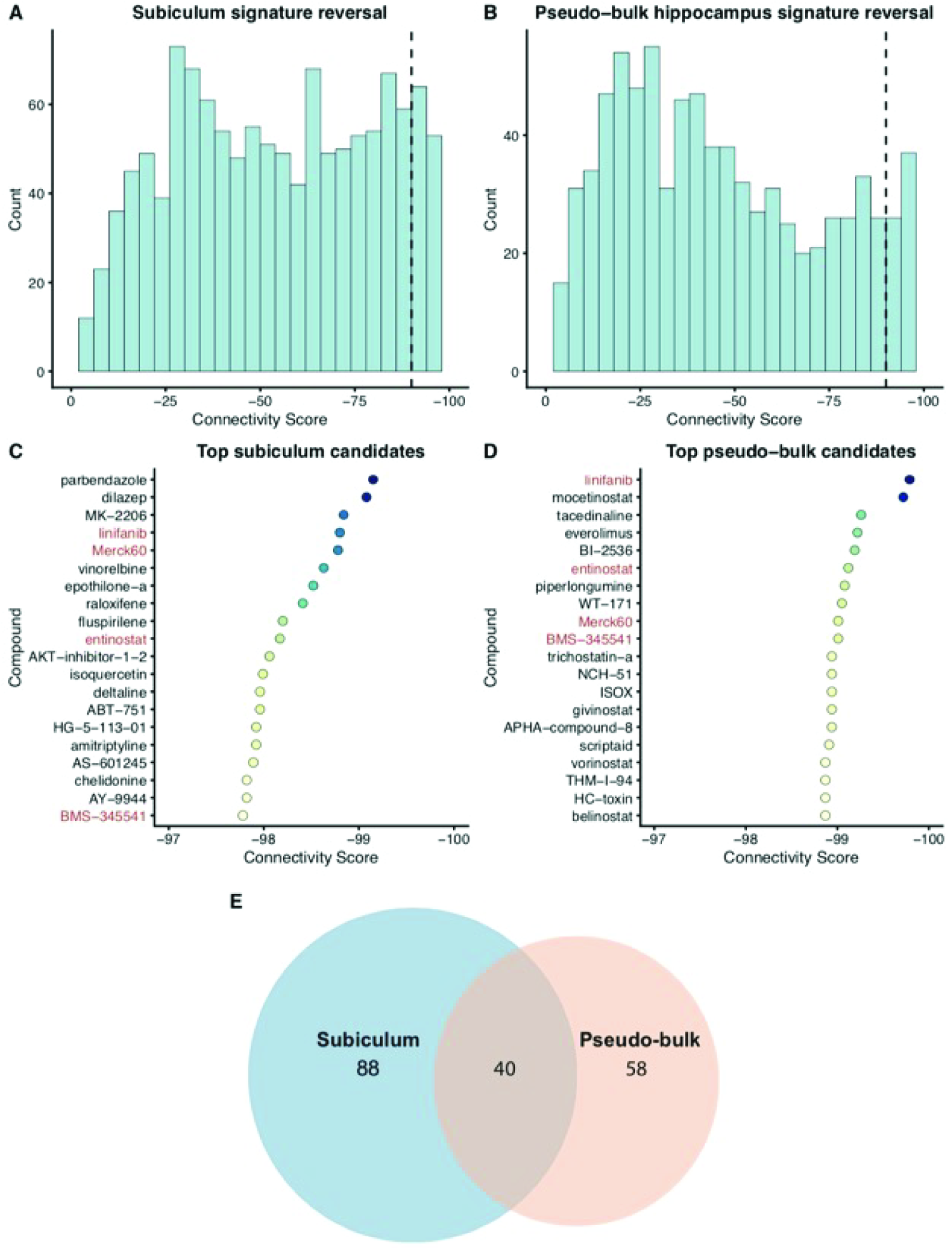

Supplement: S10 Fig — (A, B) Distribution of Connectivity Scores for the subiculum-derived and pseudo-bulk derived disease expression signatures. (C, D) Top 20 compounds predicted to reverse the subiculum and pseudo-bulk signatures. Compounds in red represent compounds prioritized for both signatures. (E) Venn Diagram depicting the overlap between all compounds achieving a Connectivity Score less than -90 for the subiculum and pseudo-bulk signatures. (TIF) [file pgen.1010952.s010.tif]
