# Supplementary material for: Neurodevelopmental deficits and cell-type-specific transcriptomic perturbations in a mouse model of HNRNPU haploinsufficiency
Source: PLoS Genet. 2023 Oct 2;19(10):e1010952. doi: 10.1371/journal.pgen.1010952 (PMC10569524; doi:10.1371/journal.pgen.1010952)
Supplement: S1 Table — (PDF) [file pgen.1010952.s011.pdf]

cDNA change (NM_031844.2)	Amino Acid change	Mutation Type	Reference
c.16delins	p.Val6Ilefs*4	PPT	[1]
c.23del	p.Val8Glufs*4	PPT	[2]
c.418G>A	p.Glu140Lys	Missense	[2]
c.511C>T	p.Gln171*	PPT	[3]
c.523C>T	p.Gln175*	PPT	[4]
c.651_660del	p.Gly218Alafs*118	PPT	[5]
c.692-1G>A	p.?	Splice site	[1]
c.817C>T	p.Gln273*	PPT	[4]
c.960G>A	p.Trp320*	PPT	[2]
c.970A>G	p.Arg324Gly	Missense	[4]
c.1089G>A	p.Trp363*	PPT	[5]
c.1117+1G>A	p.?	Splice site	[2]
c.1132T/C	p.Ser378Pro	Missense	[4]
c.1424_1425insTC	p.Ile476Profs*7	PPT	[2]
c.1615-1G>A	c.1615-1G>A	Splice site	[6]
c.1626_1627insA	p.Lys543*	PPT	[2]
c.1664del	p.Leu555Argfs*51	PPT	[2]
c.1681C>T	p.Gln561*	PPT	[1]
c.1681del	p.Gln561Serfs*45	PPT	[1]
c.1714C>T	p.Arg572*	PPT	[5]
c.1744-4_1749del	p.?	Splice site	[7]
c.1868dup	p.Glu624Argfs*24	PPT	[1]
c.2270_2271del	p.Pro757Argfs*7	PPT	[5]
c.2299_2302del	p.Asn767Glufs*66	PPT	[1]
c.2425-3C>A	p.?	Splice site	[1]
c.2471_2472delinsGA	p.Tyr824*	PPT	[8]

1. Depienne C, Nava C, Keren B, Heide S, Rastetter A, Passemard S, et al. Genetic and phenotypic dissection of 1q43q44 microdeletion syndrome and neurodevelopmental phenotypes associated with mutations in ZBTB18 and HNRNPU. Hum Genet. 2017;136: 463–479. doi:10.1007/s00439-017-1772-0

2. Yates TM, Vasudevan PC, Chandler KE, Donnelly DE, Stark Z, Sadedin S, et al. De novo mutations in HNRNPU result in a neurodevelopmental syndrome. Am J Med Genet A. 2017;173: 3003–3012. doi:10.1002/ajmg.a.38492

3. Hamdan FF, Srour M, Capo-Chichi J-M, Daoud H, Nassif C, Patry L, et al. De novo mutations in moderate or severe intellectual disability. PLoS genetics. 2014;10: e1004772. doi:10.1371/journal.pgen.1004772

4. Bramswig NC, Lüdecke H-J, Hamdan FF, Altmüller J, Beleggia F, Elcioglu NH, et al. Heterozygous HNRNPU variants cause early onset epilepsy and severe intellectual disability. Hum Genet. 2017;136: 821–834. doi:10.1007/s00439-017-1795-6

5. Leduc MS, Chao H-T, Qu C, Walkiewicz M, Xiao R, Magoulas P, et al. Clinical and molecular characterization of de novo loss of function variants in HNRNPU. Am J Med Genet A. 2017;173: 2680–2689. doi:10.1002/ajmg.a.38388

6. Need AC, Shashi V, Hitomi Y, Schoch K, Shianna KV, McDonald MT, et al. Clinical application of exome sequencing in undiagnosed genetic conditions. J Med Genet. 2012;49: 353–361. doi:10.1136/jmedgenet-2012-100819

7. Epi4K Consortium, Epilepsy Phenome/Genome Project, Allen AS, Berkovic SF, Cossette P, Delanty N, et al. De novo mutations in epileptic encephalopathies. Nature. 2013;501: 217–221. doi:10.1038/nature12439

8. Carvill GL, Heavin SB, Yendle SC, McMahon JM, O'Roak BJ, Cook J, et al. Targeted resequencing in epileptic encephalopathies identifies de novo mutations in CHD2 and SYNGAP1. Nat Genet. 2013;45: 825–830. doi:10.1038/ng.2646
